# Gene expression profiling reveals potential prognostic biomarkers associated with the progression of heart failure

**DOI:** 10.1186/s13073-015-0149-z

**Published:** 2015-03-14

**Authors:** Agata Maciejak, Marek Kiliszek, Marcin Michalak, Dorota Tulacz, Grzegorz Opolski, Krzysztof Matlak, Slawomir Dobrzycki, Agnieszka Segiet, Monika Gora, Beata Burzynska

**Affiliations:** Institute of Biochemistry and Biophysics, Polish Academy of Sciences, Warsaw, Poland; 1st Chair and Department of Cardiology, Medical University of Warsaw, Warsaw, Poland; Department of Cardiology and Internal Diseases, Military Institute of Medicine, Warsaw, Poland; Department of Cardiac Surgery, Medical University of Bialystok, Bialystok, Poland; Department of Invasive Cardiology, Medical University of Bialystok, Bialystok, Poland; Faculty of Mathematics, Informatics and Mechanics, University of Warsaw, Warsaw, Poland; 1st Medical Faculty, Medical University of Warsaw, Warsaw, Poland

## Abstract

**Background:**

Heart failure (HF) is the most common cause of morbidity and mortality in developed countries. Here, we identify biologically relevant transcripts that are significantly altered in the early phase of myocardial infarction and are associated with the development of post-myocardial infarction HF.

**Methods:**

We collected peripheral blood samples from patients with ST-segment elevation myocardial infarction (STEMI): n = 111 and n = 41 patients from the study and validation groups, respectively. Control groups comprised patients with a stable coronary artery disease and without a history of myocardial infarction. Based on plasma NT-proBNP level and left ventricular ejection fraction parameters the STEMI patients were divided into HF and non-HF groups. Microarrays were used to analyze mRNA levels in peripheral blood mononuclear cells (PBMCs) isolated from the study group at four time points and control group. Microarray results were validated by RT-qPCR using whole blood RNA from the validation group.

**Results:**

Samples from the first three time points (admission, discharge, and 1 month after AMI) were compared with the samples from the same patients collected 6 months after AMI (stable phase) and with the control group. The greatest differences in transcriptional profiles were observed on admission and they gradually stabilized during the follow-up. We have also identified a set of genes the expression of which on the first day of STEMI differed significantly between patients who developed HF after 6 months of observation and those who did not. *RNASE1*, *FMN1*, and *JDP2* were selected for further analysis and their early up-regulation was confirmed in HF patients from both the study and validation groups. Significant correlations were found between expression levels of these biomarkers and clinical parameters. The receiver operating characteristic (ROC) curves indicated a good prognostic value of the genes chosen.

**Conclusions:**

This study demonstrates an altered gene expression profile in PBMCs during acute myocardial infarction and through the follow-up. The identified gene expression changes at the early phase of STEMI that differentiated the patients who developed HF from those who did not could serve as a convenient tool contributing to the prognosis of heart failure.

**Electronic supplementary material:**

The online version of this article (doi:10.1186/s13073-015-0149-z) contains supplementary material, which is available to authorized users.

## Background

Genome-wide gene expression profiling is an extensively used strategy for discovering new potential biomarkers for diagnosis/prediction of disease severity [1,2] and identification of novel drug targets [3]. Transcriptome analysis has been applied successfully to numerous complex diseases including cardiovascular disorders [4,5].

Coronary heart disease (CHD) is one of the major causes of heart failure (HF), the predominant cause of morbidity and mortality in developed countries. HF is a major public health concern whose incidence is continuing to increase. While advances in the management of HF have improved patient outcomes, it remains the leading hospital admission diagnosis in elderly patients and carries a 5-year mortality rate as high as 50% [6]. The HF prevalence in the general population in the developed countries is estimated to be in the range of 0.4% to 2% [7]. Thus, it can be assumed that 6.5 to 10 million patients in Europe may experience HF. The ageing of the general population and the advances in the treatment of cardiovascular disease (CVD) have led to a gradual growth of the HF cohort, increasing the percentage of patients requiring hospitalization and intensive medical care.

Acute myocardial infarction (AMI) induces left ventricular (LV) remodeling, a process that can influence ventricular functions and survival outcomes. LV remodeling is directly implicated in the post-infarction development of ventricular dilatation, a predictive sign for a future HF [8]. The progression to HF after AMI is multifactorial and depends on the extent of the myocardial damage at the time of the index event, recurrent ischemia and the development of myocardial stunning and hibernation, LV remodeling, and chronic neuroendocrine stimulation. Robust early prediction of LV remodeling and the development of HF after AMI is challenging and may potentially be improved by the identification of novel transcriptional biomarkers associated with these processes [9].

Several biomarkers are known to be associated with LV remodeling and the development of HF [10]. Among the most important ones are natriuretic peptides, in particular B-type natriuretic peptide (BNP) and N-terminal pro-brain natriuretic peptide (NT-proBNP). They have a fair prognostic value in patients with acute coronary syndromes in terms of the development of heart failure [11,12]. Their diagnostic/prognostic usefulness is enhanced by other biomarkers such as troponin I and C-reactive protein (CRP) [13]. However, these biomarkers exhibit elevated levels also in patients with renal failure, primary aldosteronism, congestive heart failure, and thyroid disease [14]. There is, therefore, a need for novel, more reliable, predictive biomarkers specific for the development of HF.

The main aims of the present study were: (1) to establish alterations in gene expression patterns in leukocytes associated with acute MI and through a follow-up; and (2) to identify distinct biomarkers that correlate with HF development.

## Methods

### Patients

Between the years 2010 and 2013, we studied consecutive patients with ST elevation myocardial infarction (STEMI) who were indicated for direct percutaneous coronary interventions (PCI). The study group comprised n = 111 patients, who were admitted to the First Chair and Department of Cardiology of the Medical University of Warsaw, and the validation group comprised n = 41 patients admitted to the Department of Cardiac Surgery and Department of Invasive Cardiology, Medical University of Bialystok. All the patients underwent coronary angiography and angioplasty of the infarct-related artery. Pharmacological treatment was according to current guidelines [15]. Participation in the study had no influence on the pharmacological treatment and the procedures the patients underwent. Patients from the study and validation groups underwent echocardiography and left ventricular ejection fractions (LVEF) were calculated during hospitalization, 1 month, and 6 months after STEMI. Plasma NT-proBNP level was measured on the first day of AMI (admission), after 4 to 6 days (discharge), and 6 months after AMI using the Roche Diagnostics Elecsys® proBNP Immunoassay (Roche, Mannheim, Germany). The withdrawal rate was similar in both groups: for the study group n = 83 patients completed the study (75%), and for the validation group n = 32 (78%).

The aim of our analysis was to find expression of genes linked specifically with MI and HF, not with coronary artery disease (CAD). To exclude genes linked with CAD we decided to take as a control group patients with stable CAD, not healthy controls. The blood samples were collected from n = 46 patients (control I for study group) and n = 21 patients (control II for validation group) with CAD proven using coronary angiography (at least one stenosis exceeding 50% or coronary angioplasty of a previous coronary artery bypass graft) or non-invasive tests (positive exercise test), and no history of myocardial infarction. NT-proBNP and LVEF were measured in the control group only on admission.

The STEMI patients from the study group who had a myocardial infarction for the first time and volunteered for a control visit 6 months after AMI were divided on the basis of plasma NT-proBNP level and LVEF (measured 6 months after AMI) into four equal groups. Among the patients from the first (Q1) and fourth (Q4) quartiles we selected only those who had: a high level of NT-proBNP and low LVEF 6 months after AMI (HF group, n = 9 patients), or low level of NT-proBNP and high LVEF 6 months after AMI (non-HF group, n = 8 patients).

The validation group comprised patients who had a first myocardial infarction and volunteered for a control visit 6 months after AMI. They were divided based on the LVEF measurements into four equal groups. Patients from the first (Q1) and fourth (Q4) quartile (low LVEF 6 months after AMI, HF patients, n = 7 and high LVEF 6 months after AMI, non-HF patients, n = 7, respectively), were studied further.

The study was approved by the local Ethics Committees of the Medical University of Warsaw and Medical University of Bialystok, and was conducted in accordance with the principles of the Declaration of Helsinki. All participants gave written informed consent.

### Blood collection and RNA isolation

Whole blood samples were collected from the patients at four time points: admission, discharge, after 1 month, and after 6 months.

For the study group and control group I, peripheral blood mononuclear cells (PBMCs) were isolated within 1 h of collection using BD Vacutainer® CPT™ Cell Preparation Tubes with sodium citrate (Becton, Dickinson and Co., Franklin Lakes, NJ, USA) according to the manufacturer’s instructions. Total RNA was isolated from PBMCs with the MagNA Pure Compact System (Roche Diagnostics GmbH, Germany) following the manufacturer’s recommendations.

For the validation and control group II, the PAXgene Blood RNA system was used as it stabilizes RNA immediately after sample collection and enables the storage of samples for a relatively long period of time. Whole blood was collected directly into PAXgene Blood RNA tubes and total RNA was isolated within 24 h of collection using the PAXgene Blood RNA kit (QIAGEN, Hilden, Germany), following the manufacturer’s protocol.

RNA quantity was determined by UV absorption (Nanodrop, LabTech International, UK). The quality of RNA samples was checked using an Agilent 2100 Bioanalizer© and RNA 6000 Nano Kit (Agilent, Santa Clara, CA, USA). Samples with an RNA integrity number of 8 or above were considered suitable for use. RNA samples were stored at -80°C until further analysis.

**Figure 1:**
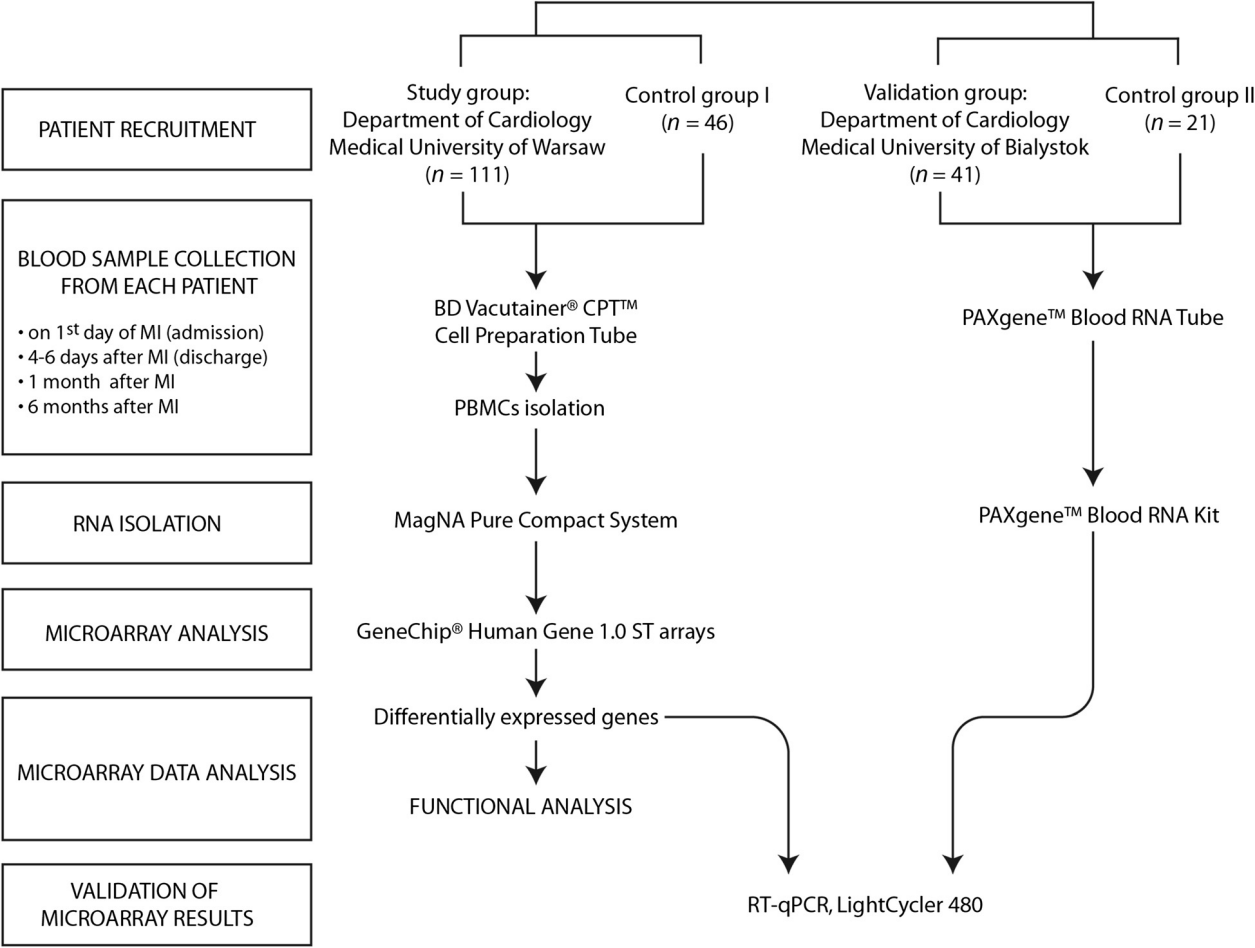
**Outline of study design.**

A schematic of patient cohorts and methodology is shown in Figure 1.

### cDNA microarrays

Preparation of labeled cDNA and hybridization to GeneChip® Human Gene 1.0 ST arrays (Affymetrix, Santa Clara, CA, USA) was performed according to the manufacturer’s instructions. Briefly, a total of 100 ng of RNA was reverse transcribed, amplified, and labeled with biotin using the GeneChip® Whole Transcript (WT) Sense Target Labeling Assay with included quality control GeneChip® Hybridization Control Kits (Affymetrix). Hybridization to the microarrays was conducted for 16 h at 45°C. After hybridization the microarrays were washed and stained on an Affymetrix GeneChip Fluidics Station 450 and scanned on an Affymetrix GCS 3000 GeneArray Scanner. The raw microarray data reported in this manuscript have been deposited in the Gene Expression Omnibus (GEO) database with the accession number GSE59867.

### Data analysis of microarrays

Quality control was conducted using the Affymetrix® Expression Console™ Software and standard Affymetrix quality metrics. Raw microarray data were background corrected, log transformed, and quantile normalized using the robust multi-array average (RMA) algorithm as implemented in the Partek® Genomics Suite™ software (Partek Inc., St. Louis, MO, USA). The fold change (FC) of gene expression ratios >1.3 and *P* <0.05 were set as significance criteria to identify genes whose expression was differentially regulated in the study group at four time points. For transcriptional profiling in the HF and non-HF groups, the significance criteria were set at FC >1.5 and *P* <0.05. Probesets lacking annotation information were removed from further analysis. Genes represented on microarrays by more than one probeset were counted only once. Thus, the number of transcripts refers to the number of unique genes.

To identify groups of functionally related genes, gene ontology (GO) analysis was performed using the AmiGO's Term Enrichment tool (version 1.8, [16]). The Ingenuity Pathway Analysis (IPA) software (Ingenuity® Systems, [17]) was used to generate molecular interaction networks and assess statistically relevant biofunctions and canonical pathways associated with the lists of differentially expressed genes.

### Quantitative real-time RT-PCR

The real-time reverse transcription-polymerase chain reaction (RT-qPCR) was used to validate the microarray results. Reverse transcription was carried out using total RNA sample (200 ng from PBMCs, 800 ng from PAX) and the QuantiTect Reverse Transcription kit (QIAGEN, Hilden, Germany) according to the manufacturer's protocol. Primer sequences and reaction conditions are provided in Additional file 1. Each sample was run in triplicate in 96-well plates using LightCycler®480 and LightCycler®480 FastStart SYBR Green I Master (Roche Diagnostics GmbH, Germany). Quantification cycles (Cq) were calculated using the second derivative method (LightCycler®480 Software, Version 1.5 provided by Roche). The fold change of gene expression levels, corrected by efficiency, was analyzed using Relative Expression Software Tool (REST 2009) [18]. The expression data were normalized to the reference gene *HPRT1*. All experiments (sample collection, preparation and storage, primer design) were performed according to the MIQE guidelines [19].

### Statistical methods

Statistical analysis was performed on R version 3.0.2 [20]. Continuous variables are presented as mean ± standard deviation, categorical variables are reported as frequencies and percentages. The distribution of continuous variables was first analyzed with the Shapiro-Wilk test of normality and then, depending on the results, the *t*-Student test, Mann-Whitney test, ANOVA or Kruskal-Wallis test was applied. Categorical variables were compared using Fisher’s exact test. The significance level was set at 0.05.

To assess the discriminatory power of each marker, a receiver operating characteristic (ROC) curve was constructed and the area under the curve (AUC) with 95% confidence interval was calculated. The cutoff value for each marker was defined as the marker fold change that corresponds to the point on the ROC curve closest to the point (0, 1). To assess monotonic association between expression levels of the biomarkers investigated and HF development indicators, a Spearman’s rank correlation coefficient and its *P* value were calculated for each biomarker’s fold change-value on admission and NT-proBNP and LVEF 6 months after AMI.

## Results

### Clinical characteristics of study groups

Patients presenting with STEMI, treated with primary percutaneous revascularization, were enrolled in this study. Their mean age ± SD was 58.8 ± 10.4 years and 61 ± 10.6 years for the study (n = 111) and the validation (n = 41) groups, respectively. The baseline demographic and clinical characteristics of the study and validation groups are summarized in Table 1. Significant differences between the two groups were observed for two parameters: hypercholesterolemia was significantly more common (*P* <0.001), and LVEF measured on admission was significantly lower (*P* <0.001) in the validation compared to the study group.

**Table 1:** **Baseline demographic and clinical characteristics of study and validation groups**

**Characteristics**	**Study group (n = 111)**	**Validation group (n = 41)**	***P*** **value**
Men	86 (77.5%)	31 (75.6%)	0.83
Women	25 (22.5%)	10 (24.4%)	0.83
Age (years)	58.8 ± 10.4	61 ± 10.6	0.277
BMI (kg/m^2^)	28.1 ± 4.7	28.9 ± 5.1	0.517
Hypertension	62 (55.9%)	28 (71.8%)	0.09
Diabetes	26 (23.4%)	6 (15.4%)	0.367
Previous MI	8 (7.2%)	3 (7.7%)	1
Smoking	54 (48.6%)	20 (51.3%)	0.853
Hypercholesterolemia	60 (54.1%)	34 (87.2%)	<0.001
AMI	54 (51.4%)	17 (43.6%)	0.456
Previous revascularization	3 (2.7%)	2 (5.1%)	0.605
Non-coronary atherosclerosis	6 (5.4%)	3 (7.7%)	0.697
NT-proBNP (pg/mL)	1,641.4 ± 3,675.3	2,913.3 ± 6,975.7	0.559
LVEF (%)	49.3 ± 8.6	42.1 ± 8.3	<0.001
Medications^a^			
Aspirin	105 (100%)	39 (100%)	NA
Clopidogrel	104 (99%)	39 (100%)	1
Beta blockers	104 (99%)	38 (97.4%)	0.47
ACE inhibitors	100 (95.2%)	39 (100%)	0.324
Statins	103 (98.1%)	39 (100%)	1
Diuretics	21 (20%)	14 (35.9%)	0.079

^a^Data were only available for n = 105 patients from the study group and n = 39 from validation group; these numbers were used to calculate percentages.

Data at admission.

Data are presented as mean value ± standard deviation or number or percentage of patients. *P* value <0.05 was considered significant.

ACE, angiotensin-converting enzyme; AMI, anterior myocardial infarction; BMI, body mass index; LVEF, left ventricular ejection fraction; MI, myocardial infarction; NA, not applicable; NT-proBNP, N-terminal pro-brain natriuretic peptide.

The characteristics of HF (n = 9) and non-HF (n = 8) patients from the study group are given in Table 2, and of the HF (n = 7) and non-HF patients (n = 7) from validation group in Table 3.

**Table 2:** **Baseline demographic and clinical characteristics of HF, non-HF patients from study group**

**Characteristics**	**HF patients (n = 9)**	**Non-HF patients (n = 8)**	***P*** **value**
Men	6 (66.7%)	7 (87.5%)	0.576
Women	3 (33.3%)	1 (12.5%)	0.576
Age (years)	60.1 ± 14.3	51.8 ± 7.2	0.147
BMI (kg/m^2^)	26.8 ± 3.1	25.6 ± 1.6	0.323
Hypertension	3 (33.3%)	1 (12.5%)	0.576
Diabetes	2 (22.2%)	1 (12.5%)	>0.999
Previous MI	0 (0%)	0 (0%)	NA
Smoking	3 (33.3%)	5 (62.5%)	0.347
Hypercholesterolemia	5 (55.6%)	4 (50%)	>0.999
AMI	8 (88.9%)	3 (42.9%)	0.106
NT-proBNP (pg/mL)^a^	918.3 ± 848.5	62 ± 14.1	<0.001
LVEF (%)^a^	39.3 ± 8.4	66.8 ± 1.9	0.001
Medications			
Aspirin	9 (100%)	8 (100%)	NA
Clopidogrel	8 (88.9%)	8 (100%)	>0.999
Beta blockers	9 (100%)	8 (100%)	NA
ACE inhibitors	9 (100%)	8 (100%)	NA
Statins	9 (100%)	8 (100%)	NA
Diuretics	7 (77.8%)	1 (12.5%)	0.015

^a^NT-proBNP, LVEF measured 6 months after AMI.

Data are presented as mean value ± standard deviation or number or percentage of patients. *P* value <0.05 was considered significant.

ACE, angiotensin-converting enzyme; AMI, anterior myocardial infarction; BMI, body mass index; LVEF, left ventricular ejection fraction; MI, myocardial infarction; NA, not applicable; NT-proBNP, N-terminal pro-brain natriuretic peptide.

**Table 3:** **Baseline demographic and clinical characteristics of HF and non-HF patients from validation group**

**Characteristics**	**HF patients (n = 7)**	**Non-HF patients (n = 7)**	***P*** **value**
Men	7 (100%)	7 (100%)	NA
Women	0 (0%)	0 (0%)	NA
Age (years)	59.7 ± 9.4	56.7 ± 13.2	0.634
BMI (kg/m^2^)	30 ± 3.7	26.1 ± 4.2	0.092
Hypertension	5 (71.4%)	5 (71.4%)	1
Diabetes	2 (28.6%)	0 (0%)	0.462
Previous MI	0 (0%)	0 (0%)	NA
Smoking	3 (42.9%)	5 (71.4%)	0.592
Hypercholesterolemia	5 (71.4%)	5 (71.4%)	1
AMI	3 (42.9%)	2 (28.6%)	1
NT-proBNP (pg/mL)^a^	1,960.9 ± 3,421.9	119.4 ± 118.9	0.002
LVEF (%)^a^	28.9 ± 6.3	57.7 ± 4.7	<0.001
Medications			
Aspirin	7 (100%)	7 (100%)	NA
Clopidogrel	7 (100%)	7 (100%)	NA
Beta blockers	7 (100%)	6 (85.7%)	1
ACE inhibitors	7 (100%)	7 (100%)	NA
Statins	7 (100%)	7 (100%)	NA
Diuretics	5 (71.4%)	1 (14.3%)	0.103

^a^NT-proBNP, LVEF measured 6 months after AMI.

Data are presented as mean value ± standard deviation or number or percentage of patients. *P* value <0.05 was considered significant.

ACE, angiotensin-converting enzyme; AMI, anterior myocardial infarction; BMI, body mass index; LVEF, left ventricular ejection fraction; MI, myocardial infarction; NA, not applicable; NT-proBNP, N-terminal pro-brain natriuretic peptide.

The baseline demographic and clinical characteristics of the control groups are shown in Additional file 2.

### Gene expression profiling at different time points following AMI

To determine gene expression profiles and their possible changes during the recovery from myocardial infarction, we performed a transcriptome analysis in PBMCs isolated from n = 111 AMI patients (study group) at four time points, and from n = 46 CAD patients with no history of MI (control group I). Samples from the first three time points (admission, discharge, and 1 month after AMI) were compared with the samples from the same patients collected 6 months after AMI (stable phase), which minimized the effects of inter-patient variability. Additionally, a comparison between AMI samples and samples from the control group was performed to identify genes shared between the comparisons. We identified 197 transcripts (153 up- and 44 downregulated) that were differentially expressed on admission compared to 6 months after AMI (Additional file 3). Among them, 77 transcripts were also found to differ between admission and the control group (Additional file 4). These transcripts comprise an expression signature of the acute phase of MI. On discharge 41 transcripts (40 up- and 1 downregulated) were differentially expressed compared to 6 months after AMI (Additional file 5). Notably, most of them encoded immunoglobulins. Similarly, transcripts involved mainly in immune response were found to differ between discharge and the control group. Twenty-seven differentially expressed genes were common to both comparisons (Additional file 6). One month after AMI only two transcripts, both encoding immunoglobulins (*IGJ* and *IGKVI-33*), were upregulated compared to 6 months after AMI (Additional file 7). *IGJ* was also found to be expressed differentially between 1 month after AMI and the control group.

**Figure 2:**
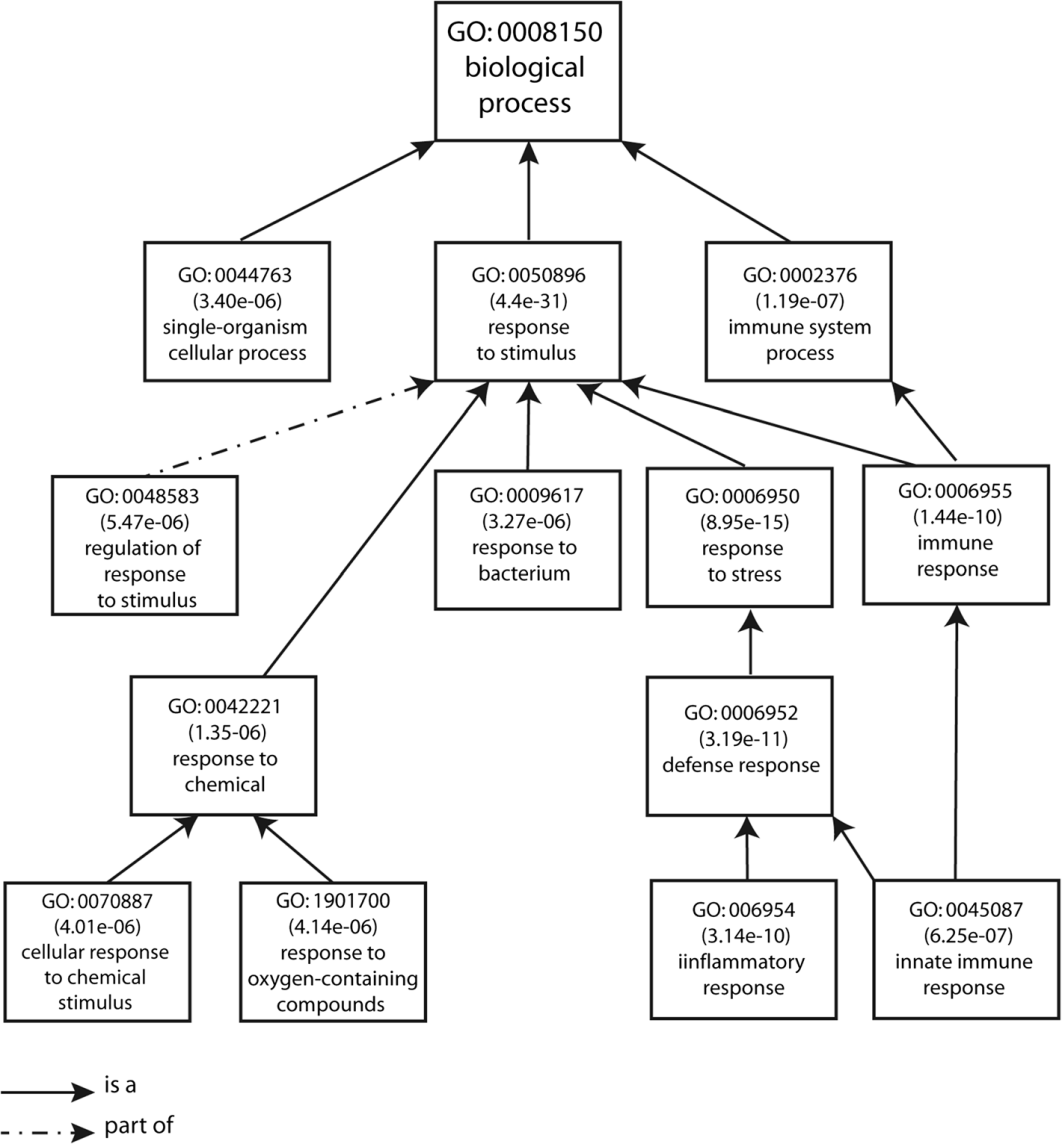
**Top enriched GO categories among genes differentially expressed on admission versus 6 months after AMI.**

**Figure 3:**
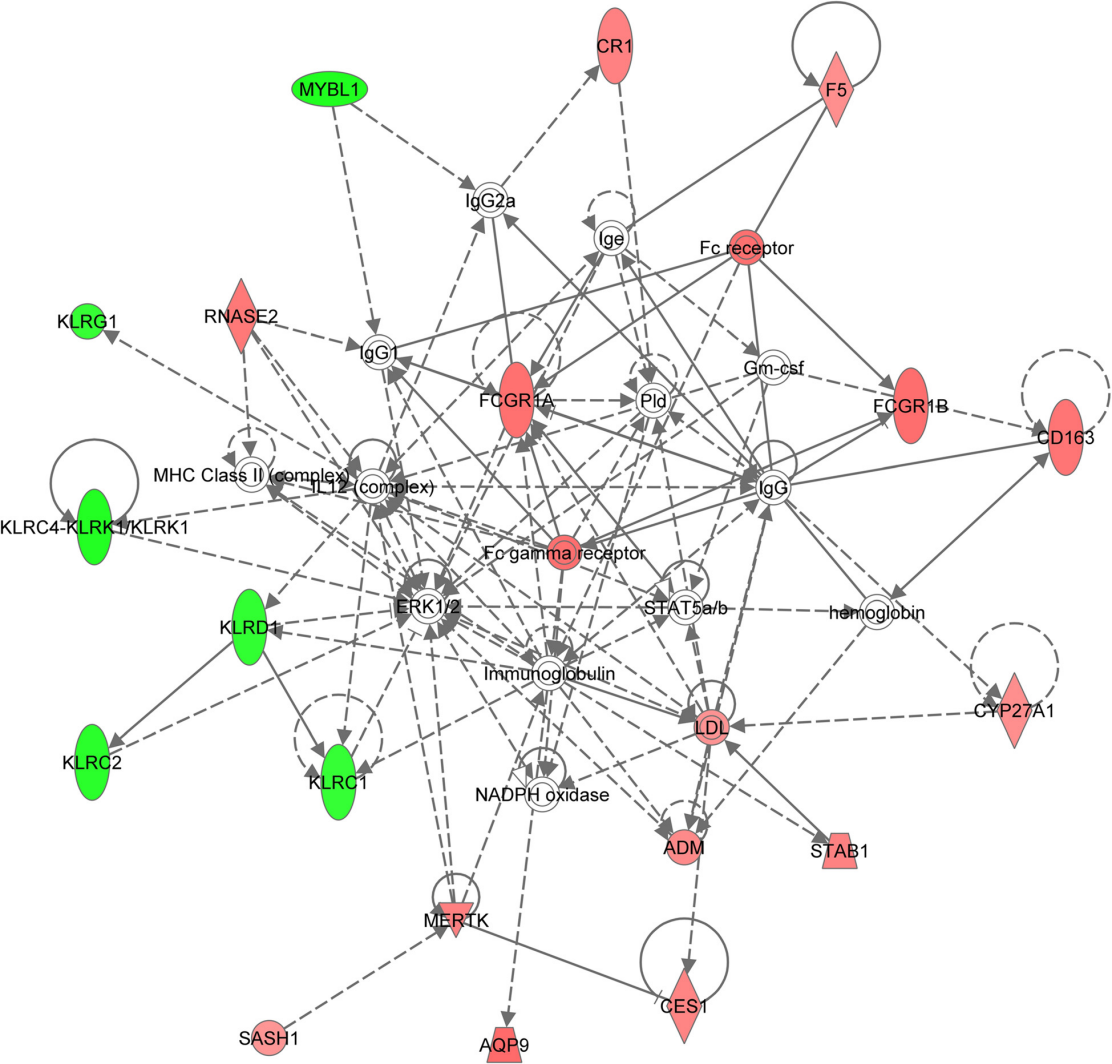
**Top scoring interaction network for 77 differentially expressed transcripts in the acute phase of MI.** The network is classified as ‘Cell-To-Cell Signaling and Interaction, Hematological System Development and Function, Immune Cell Trafficking’. Genes or gene products are represented as nodes, and the biological relationship between two nodes is represented as solid line (direct relationships) or dotted line (indirect relationship). Upregulated and downregulated genes are shown in red and green shading, respectively, with color intensity related to the fold change in expression.

A functional category analysis was carried out for the transcripts differentially expressed in the acute phase of MI (77 transcripts common to both analyses: admission versus 6 months after AMI and admission versus control). The gene ontology analysis using AmiGO revealed that the molecular functions of these transcripts were mainly associated with ‘protein binding’. The cellular component classification showed that most of the differently expressed genes were involved in both the extracellular region and the cell membrane. The most significant biological processes were ‘response to stimulus and stress’ , ‘immune system process (response and defense)’ , and ‘single-organism cellular process’ (Figure 2). The 77 transcripts were also subjected to an interaction network analysis using the IPA software. The results showed that 19 genes (13 upregulated and 6 downregulated) were involved in the top scoring network associated with ‘Cell-To-Cell Signaling and Interaction, Hematological System Development and Function, Immune Cell Trafficking’ (Figure 3).

Our previous pilot analysis on a cohort of n = 28 patients identified 24 genes as an expression signature of the acute phase of MI [21]. It should be pointed out that the differential expression of all those 24 transcripts was confirmed in the current study on a substantially larger group of patients when the reference were samples from the same patients collected 6 months after AMI (Additional file 3). When compared with the independent group of patients with no history of MI (control group), 18 transcripts showed differential expression.

To confirm the robustness of the microarray results in an independent cohort, mRNA levels of selected four signature genes: aquaporin 9 (*AQP9*), family with sequence similarity 20, member A (*FAM20A*), and peroxisome proliferator-activated receptor gamma (*PPARG*), suppressor of cytokine signaling 3 (*SOCS3*) were quantified by RT-qPCR using samples from the validation group (n = 41) collected at the same four time points after AMI and from control group II (n = 21). The greatest differences in expression were observed in the acute phase after MI (admission vs. 6 months and admission vs. control group II), and for all four genes the direction and magnitude of the change agreed well with the microarray data, as depicted in Tables 4 and 5. At the next two time points (discharge and 1 month after AMI) the differences in gene expression relative to that 6 months after AMI and to control group II were less prominent, except for *FAM20A*. We stress here that the gene expression changes detected using microarray analysis in PBMCs in the study group were validated here using a different technique, an independent cohort, and whole blood as the source of RNA. This shows that the choice of material (whole blood vs. PBMCs) and analytical method (microarrays vs. RT-qPCR) does not affect the obtained results, indicating the robustness of the proposed approach.

**Table 4:** **RT-qPCR results for selected genes in validation group at different time points after AMI**

**Gene symbol**	**Admission vs. 6 months**				**Discharge vs. 6 months**				**1 month vs. 6 months**			
	**Microarray**		**RT-qPCR**		**Microarray**		**RT-qPCR**		**Microarray**		**RT-qPCR**	
	**Study group**		**Validation group**		**Study group**		**Validation group**		**Study group**		**Validation group**	
	**Fold change**	***P*** **value**	**Fold change**	***P*** **value**	**Fold change**	***P*** **value**	**Fold change**	***P*** **value**	**Fold change**	***P*** **value**	**Fold change**	***P*** **value**
*AQP9*	1.862	***	1.580	***	1.265	***	1.158	ns	1.047	ns	-1.046	ns
*FAM20A*	1.908	***	4.283	***	1.547	***	3.104	***	1.082	ns	1.015	ns
*PPARG*	1.613	***	2.213	***	1.144	***	1.193	ns	-1.012	ns	-1.360	*
*SOCS3*	2.551	***	1.794	***	1.415	***	1.177	ns	1.064	ns	-1.144	ns

Statistical significance: *P <0.05. ***P <0.001.

Results were normalized to *HPRT1*.

ns, non-significant.

**Table 5:** **RT-qPCR results for selected genes in validation group in comparison with control group**

**Gene symbol**	**Admission vs. control group**				**Discharge vs. control group**				**1 month vs. control group**			
	**Microarray**		**RT-qPCR**		**Microarray**		**RT-qPCR**		**Microarray**		**RT-qPCR**	
	**Study group**		**Validation group**		**Study group**		**Validation group**		**Study group**		**Validation group**	
	**Fold change**	***P*** **value**	**Fold change**	***P*** **value**	**Fold change**	***P*** **value**	**Fold change**	***P*** **value**	**Fold change**	***P*** **value**	**Fold change**	***P*** **value**
*AQP9*	1.622	***	1.298	*	1.102	ns	-1.055	ns	-1.095	ns	-1.190	ns
*FAM20A*	1.704	***	4.580	***	1.382	*	3.288	***	-1.034	ns	1.165	ns
*PPARG*	1.494	***	1.753	**	1.059	ns	1.790	**	-1.093	ns	-1.287	ns
*SOCS3*	1.840	***	1.694	***	1.020	ns	1.097	ns	-1.303	*	-1.305	*

Statistical significance: *P <0.05. **P <0.01. ***P <0.001.

Results were normalized to *HPRT1*.

ns, non-significant.

### Gene expression profiling in HF and non-HF groups

**Figure 4:**
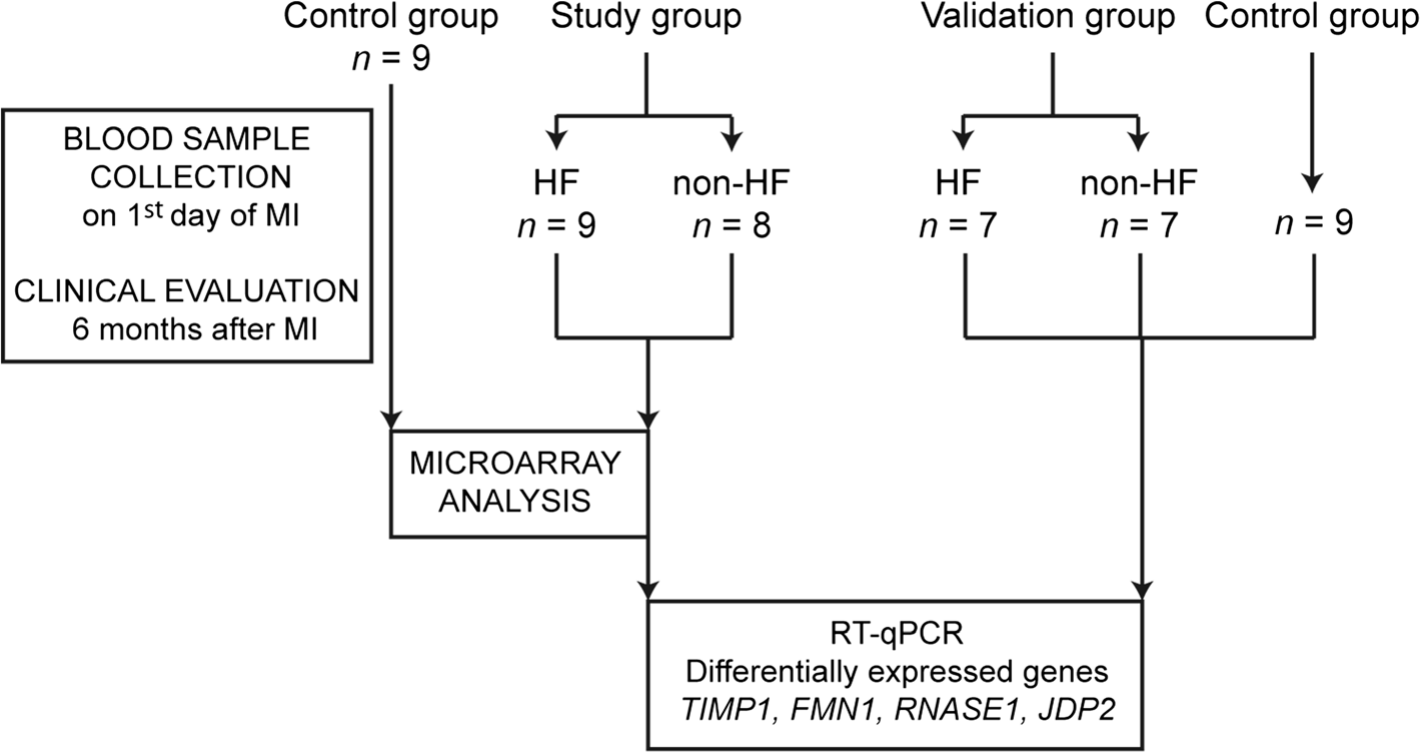
**Outline of selection and distribution of patients from HF, non-HF, and control groups.**

We attempted to identify transcripts whose differential expression on the first day of myocardial infarction predicted which patients would develop symptoms of HF during the 6 months of follow-up (Figure 4). For this purpose, we compared the microarray results for samples collected on admission for the HF group versus the non-HF group. A total of 127 transcripts showed significant difference between the two groups (49 were up- and 77 downregulated in HF patients) (Additional file 8).

We selected from control group I (with proven CAD and no history of myocardial infarction) a random age-matched subgroup of patients (n = 9) to perform an additional microarray analysis. We compared the HF group and the non-HF group with this control group and from this comparison, we identified 256 transcripts (155 up- and 101 downregulated) differentially expressed in HF patients on admission and 58 transcripts (27 up- and 31 downregulated) in non-HF patients on admission (Additional files 9 and 10). Twenty transcripts were regulated similarly in the HF and non-HF patients, 236 transcripts (138 up- and 98 downregulated) were unique to the HF group, and 38 transcripts (10 up- and 28 downregulated) to the non-HF group.

Functional analysis of the HF versus non-HF dataset revealed that processes associated with immune response and cell death were predominantly affected in HF patients. ‘Immune system process’ , ‘apoptotic process’ , and ‘programmed cell death’ were significantly overrepresented biological processes identified by AmiGO. The IPA analysis indicated that three highest scoring networks are associated with ‘humoral immune response’ , ‘immunological disease’ , and ‘inflammatory disease’. The most significantly affected canonical pathways were ‘iCOS-iCOSL signaling in T helper cells’ , ‘IL-6 signaling’ , and ‘T helper cell differentiation’. In the ‘Molecular and Cellular Functions’ category ‘cell death and survival’ , ‘cell-to-cell signaling and interaction’ , and ‘cellular growth and function’ were the most affected.

Based on individual microarray intensities for selected genes (Additional file 11) and a systemic literature search we selected for further studies four genes most significantly differentiating the HF from non-HF patients: formin 1 (*FMN1*), Jun dimerization protein 2 (*JDP2*), ribonuclease, RNase A family, 1 (pancreatic) (*RNASE1*), and TIMP metallopeptidase inhibitor 1 (*TIMP1*). The selected biomarkers are listed in Additional file 8. Changes in their expression levels were validated using quantitative RT-PCR on the HF and non-HF samples from the study and validation groups. The results corroborated the microarray data. For the RT-qPCR validation we used random age-matched subgroups of patients with proven CAD but no MI (n = 9 in each), selected from the control groups I and II. Evaluation of the HF and non-HF patients versus the appropriate control groups by RT-qPCR did not confirm selective overexpression of *TIMP1* in HF patients from the study group. Thus, *TIMP1* was excluded from further analyses as a potential prognostic biomarker. In the HF patients we observed greater changes in gene expression of the selected biomarkers than in non-HF patients (Table 6).

**Table 6:** **RT-qPCR results for selected genes in HF, non-HF, and control groups on admission**

**Gene symbol**	**HF vs. non-HF**						**HF vs. control group**						**Non-HF vs. control group**					
	**Microarray**		**RT-qPCR**		**RT-qPCR**		**Microarray**		**RT-qPCR**		**RT-qPCR**		**Microarray**		**RT-qPCR**		**RT-qPCR**	
	**Study group**		**Study group**		**Validation group**		**Study group**		**Study group**		**Validation group**		**Study group**		**Study group**		**Validation group**	
	**Fold change**	***P*** **value**	**Fold change**	***P*** **value**	**Fold change**	***P*** **value**	**Fold change**	***P*** **value**	**Fold change**	***P*** **value**	**Fold change**	***P*** **value**	**Fold change**	***P*** **value**	**Fold change**	***P*** **value**	**Fold change**	***P*** **value**
*FMN1*	2.765	***	2.173	*	1.843	*	3.081	***	2.659	*	3.207	*	1.114	ns	1.223	ns	1.641	ns
*JDP2*	1.825	***	1.823	**	1.506	*	1.597	**	2.548	***	2.893	***	-1.142	ns	1.398	**	1.933	**
*RNASE1*	2.044	**	3.791	**	3.007	*	2.612	***	5.014	***	6.362	***	1.278	ns	1.323	ns	2.677	*
*TIMP1*	1.523	**	1.498	**	1.681	**	1.421	**	-1.055	ns	2.250	**	-1.072	ns	-1.580	**	1.346	ns

Statistical significance: *P <0.05. **P <0.01. ***P <0.001.

Results were normalized to *HPRT1*.

ns, non-significant.

**Figure 5:**
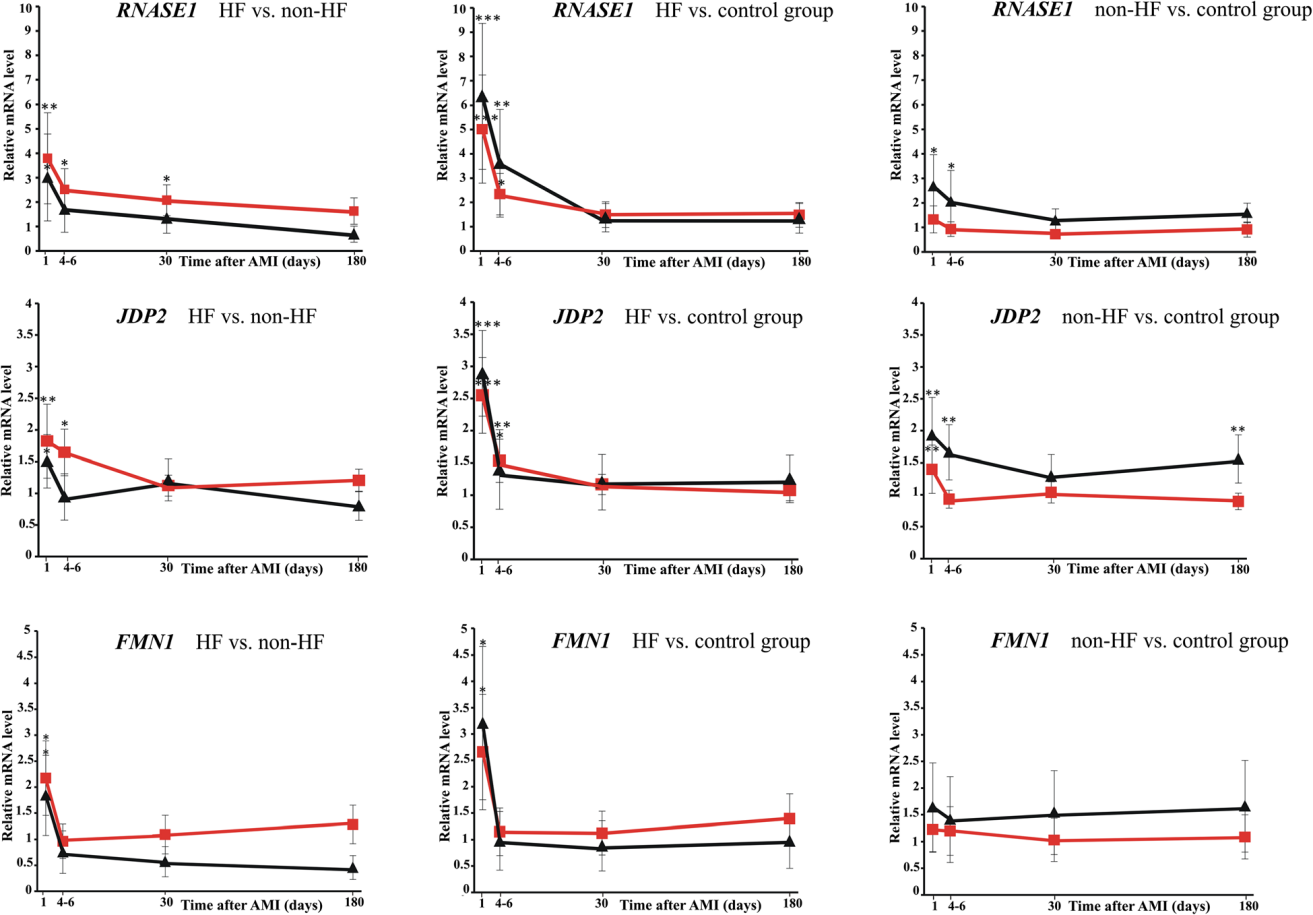
**Gene changes over time for investigated HF biomarkers in study and validation groups.** Gene expression changes were investigated in HF, non-HF, and control groups at different time points after AMI (on day 1, 4 to 6 days, 1 month, and 6 months) using RT-qPCR. Red line: study group, black line: validation group. Data represent gene expression ratio ± standard error. The error bars are absent when smaller than the size of the symbols. Statistical significance: *P <0.05; **P <0.01; ***P <0.001.

We measured gene expression levels of *RNASE1*, *JDP2*, and *FMN1* at the other time points after AMI (4 to 6 days, 1 month, 6 months) in HF, non-HF, and control groups from the study and validation groups (Figure 5). In the comparison of HF versus non-HF patients and HF versus control groups the expression level of selected biomarkers was significantly elevated on admission both in the study and validation groups. In the next time points after AMI we observed a gradual decrease in expression between admission and 1 month, and stabilization between 1 month and 6 months. In the comparison of non-HF versus control groups we noted only minor changes in the expression of the selected transcripts.

### *FMN1*, *JDP2*, and *RNASE1* as potential prognostic biomarkers associated with the progression of heart failure

To determine the relationship between the level of expression of *FMN1*, *JDP2*, and *RNASE1* upon AMI and HF development Spearman’s rank correlation coefficients were calculated for the gene’s expression fold change on admission and NT-proBNP and LVEF 6 months after AMI in the study and validation groups (Table 7). The results showed a statistically significant moderate positive monotonic correlation between each biomarker’s fold-change value on admission and the NT-proBNP level 6 months after AMI. A statistically significant negative monotonic association between *RNASE1* and *FMN1* fold-change value on admission and LVEF 6 months after AMI was also found, and a non-significant tendency for *JDP2*. Thus, an increase in the biomarkers’ fold-change value on admission is related to an increase in the NT-proBNP and a decrease in the LVEF 6 months after AMI.

**Table 7:** **Spearman’s rank correlation coefficients of selected biomarkers fold-change and NT-proBNP and LVEF values**

**Gene symbol**	**NT-proBNP**	**LVEF**
*FMN1*	0.601 (*P* = 0.001)	-0.468 (*P* = 0.009)
*JDP2*	0.417 (*P* = 0.023)	-0.355 (*P* = 0.051)
*RNASE1*	0.566 (*P* = 0.001)	-0.484 (*P* = 0.006)

Spearman’s rank correlation coefficients were calculated for the gene’s expression fold change on admission and NT-proBNP and LVEF 6 months after AMI in the study and validation groups.

**Figure 6:**
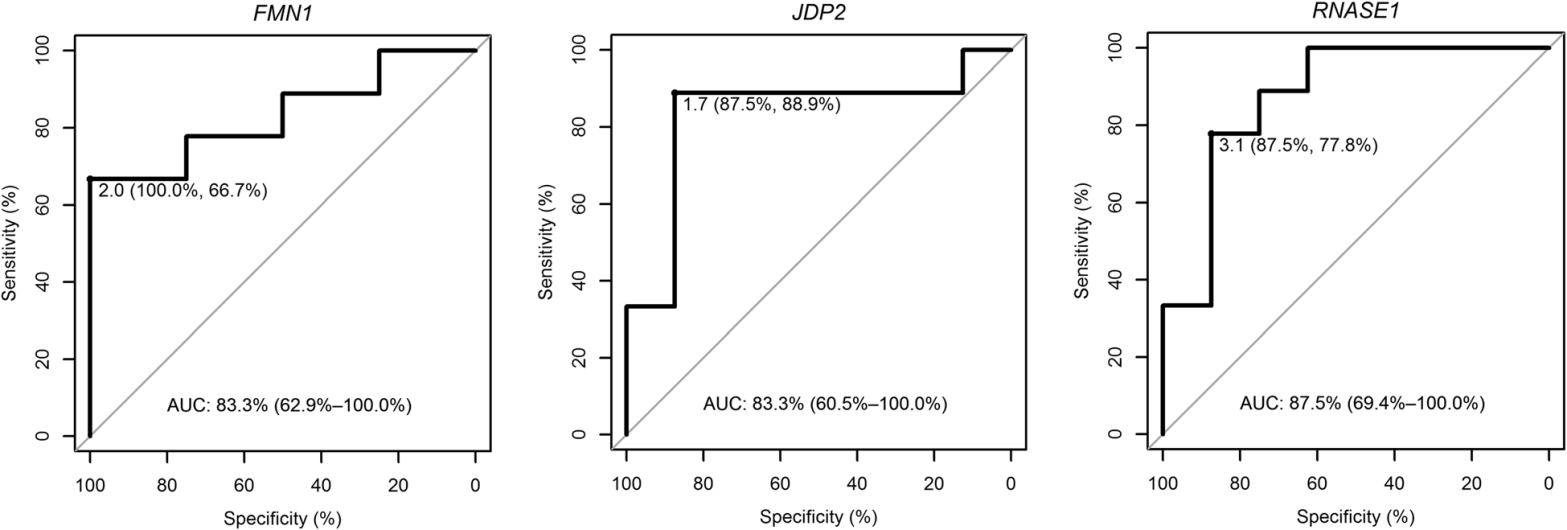
**ROC curves for** ***FMN1*** **,** ***JDP2*** **, and** ***RNASE1.*** AUC, area under the curve; ROC, receiver operating characteristic.

To investigate the value of *FMN1*, *JDP2*, and *RNASE1* as prognostic biomarkers of HF, the ROC analysis was performed on the RT-qPCR data from n = 9 patients with HF and n = 8 non-HF patients compared to the control group n = 9. The analysis showed a good predictive accuracy of those markers (Table 8 and Figure 6). For *RNASE1*, the sensitivity was 77.8% and specificity 87.5% at a cutoff value of 3.1-fold change. For *JDP2*, at a cutoff value of 1.7-fold change, these values were 88.9% and 87.5%, respectively, and for *FMN1* at a cutoff value of 2.0-fold change - 66.7% and 100%. These results indicate that the levels of expression of *FMN1*, *JDP2*, and *RNASE1* genes upon AMI are highly specific and sensitive biomarkers for predicting HF.

**Table 8:** **ROC curve analyses for** ***FMN1*** **,** ***JDP2*** **, and** ***RNASE1*** **genes**

**Gene symbol**	**AUC (%)**	**95% CI (%)**	**Cut-off value (fold change)**	**Specificity (%)**	**Sensitivity (%)**
*FMN1*	86.1	62.9-100	2	100	66.7
*JDP2*	83.3	60.5-100	1.7	87.5	88.9
*RNASE1*	87.5	69.4-100	3.1	87.5	77.8

AUC, area under the curve; CI, confidence interval; ROC, receiver operating characteristic.

## Discussion

In the present study we used gene expression profiling in PBMCs to identify biologically relevant transcripts significantly altered upon AMI and through the follow-up. We further examined the changes in the gene expression profiles unique to patients who developed HF after AMI and identified potential prognostic biomarkers associated with the post-infarction LV remodeling.

Transcriptional profiling is recently becoming a promising tool to study cardiovascular diseases [22-26]. The most important limitation for accurate investigation of the etiology and pathophysiology of HF is the necessity for heart tissue sampling. It is not always possible to perform a biopsy in a patient with myocardial dysfunction or damage. The use of blood as a surrogate tissue that can be obtained with a minimally invasive procedure is therefore an attractive alternative to cardiac biopsies. While it may be argued that transcriptomic analysis of cardiac tissues would more accurately picture the myocardial response to MI, it is accepted that several cardiovascular conditions including coronary artery disease [27] and chronic HF [28] are uniquely reflected by specific transcriptomic biosignatures in blood cells.

We have identified and characterized transcriptomic signatures and pathways associated with AMI based on gene expression analysis of PBMCs. Our previous [21] and current results indicate that in the acute phase of STEMI, dozens of genes from several pathways show altered expression. Our results suggest that PBMCs are activated in the acute phase of MI and gradually stabilized during follow-up. Numerous studies found significant roles of PBMCs in the systemic and regional inflammatory responses associated with remodeling in acute MI [29-31]. Kim *et al*. [24] presented a subset of genes associated with survival time after AMI in Caucasian subjects with CAD. They also confirmed in a large cohort that genes identified by us earlier [21] and in this study as significantly associated with AMI correspond very closely to the major component of AMI-associated gene expression. Thus, activation of the PBMCs, which reflects the magnitude of inflammation, could be related with LV remodeling and even progression of MI patients.

A patient who has had an acute MI may or may not progress to develop LV dysfunction and HF [32]. The prognosis of patients after acute coronary syndrome (ACS) largely depends on the extent of myocardial damage during the acute phase. In the cohorts of nine patients from the study group and seven from the validation group we observed that the future LV dysfunction had a specific biosignature in blood cells already in the acute phase of MI. The identified transcripts that differentiated on the first day of myocardial infarction the HF patients from the non-HF ones and the control group can serve as a novel tool contributing to early prognosis and diagnosis of post-AMI patients. This approach, validated in an independent cohort, may also be useful in searching for the molecular predisposition to the development of HF after AMI.

Our study indentified 127 transcripts significantly affected in HF patients, of which three most promising ones, *FMN1*, *JDP2*, and *RNASE1* were analyzed further. FMN1 belongs to the formin family of proteins. Ample evidence suggests that most formins are effectors of RhoA GTPase [33]. Some studies have demonstrated that RhoA/ROCK has a major role in regulating actin cytoskeleton organization, stress fiber formation, and smooth muscle cell contraction [34]. Recent studies have also demonstrated that the ROCK signaling pathway mediates the induction of cardiomyocyte hypertrophy [35]. We propose that the overexpression of *FMN1* found in our study may lead to the activation of RhoA/ROCK signaling pathways implicating further development of cardiac hypertrophy. Additionally, formins take part in the promotion of adhesion and migration of inflammatory cells - T cell and neutrophils - associated with the processes taking place in the infarct scar during acute phase MI and further left ventricular remodeling [36]. Rosado *et al*. [37] examined the expression and localization patterns of mammalian formins in cardiomyocytes and found that numerous formins localize to the sarcomere, the basic contractile unit of muscle fibers. Their distinct patterns of expression, developmental recruitment into the sarcomere, and subsarcomeric localization suggested diverse roles of formins in the development and repair of myofibrils. To the best of our knowledge, there is no evidence for a direct role of FMN1 in the pathogenesis of post-myocardial infarction HF. This is therefore a novel biomarker that could lead to new insights into the pathogenesis of HF.

The second gene/protein selected by us was JDP2, an inhibitory component of the AP-1 transcription factor. Several lines of evidence indicate an important role of the AP-1 complex in cardiac development and functioning (reviewed in [38]). This includes the induction of immune response cytokines and fetal gene expression in the failing heart. Activation of AP-1 has been reported in the early phase of myocardial infarction in the rat [39], and in both experimental and human chronic heart failure [40]. Transgenic mouse models with cardiac overexpression of JDP2 develop massive atrial dilatation and hypertrophy [41]. On the other hand, a recent study on mouse ventricular cardiomyocytes under stimulation with hypertrophy or apoptosis inducing agents has revealed protective effects of JDP2 overexpression (and thereby AP-1 inhibition) on ventricular remodeling [42]. Moreover, JDP2 has been found to induce (in association with the Nrf2/MafK complex) several detoxifying or antioxidant enzymes protecting against reactive oxygen species (ROS) [43]. It is well established that large amounts of ROS are released in myocardial ischemia and reperfusion. Given the reported data, one may hypothesize that the increased *JDP2* expression in the blood of patients with a poor ventricular outcome after MI is an adaptive response to the ischemic stress particularly profound in those patients. However, due to the diversity of the biological processes affected by JDP2, a comprehensive assessment of its role in cardiac remodeling remains to be established.

The third HF biomarker identified was RNASE1, a member of the RNase A superfamily. In addition to their ribonucleolytic activity, members of this superfamily have other biological roles like host defense [44] and angiogenesis [45]. It has been reported that human RNase1 is synthesized not only in the pancreas, but is also constitutively expressed and released by endothelial cells. Based on its presence in the plasma and serum, it has been proposed that RNase1 is expressed by vascular cells to contribute to the regulation of extracellular RNA (eRNA) [46]. The functions of eRNA in the vascular system as proinflammatory and prothrombotic agents have been shown to be neutralized by pancreatic type RNase1 [47]. eRNA released after local injury, ischemia/reperfusion or oxidant stress increases the endothelial discharge of RNase1, possibly protecting against the pro-inflammatory effects of eRNA [48]. Application of ribonuclease 1 reduced edema formation, lowered lesion volume in experimental stroke [49], and also prevented atherogenesis [50]. Several studies have employed large-scale gene expression analysis to find novel pathophysiologically important genes involved in the immune response and vascular injury. Moreover, RNASE1 was among upregulated genes identified in a multiethnic patient cohort with a history of early MI [51]. These results support our findings that increased expression of *RNASE1* can be part of the defense against pro-inflammatory effects of eRNA following massive vascular injury or tissue damage.

Our study is one of several recent attempts to identify transcriptomic biomarkers of HF development in post-AMI patients which may be helpful in understanding the pathobiology of left ventricular remodeling and in identifying biomarkers of individuals at high risk for the development of HF. *FMN1*, *JDP2*, and *RNASE1* participate in diverse processes activated under stress during injury. We postulate that the upregulation of these genes is connected with the more severe initial damage to the heart culminating later in HF. The receiver operating characteristic analysis has indicated that these three transcripts are likely to be good biomarkers with high sensitivity and specificity for HF prognosis and implicated in the course of LV remodeling. Additionally, the validation in two independent cohorts with the use of different blood collection techniques has documented a high diagnostic accuracy of the identified genes; nevertheless, further studies with larger patient groups are needed to prove unequivocally their value as biomarkers of prospective heart failure.

### Study limitations

The major limitation of this study is the relatively small patient groups with developed HF. Although the biomarkers were verified on an independent group it will be necessary to corroborate our findings on a larger cohort. The specificity and sensitivity of our biomarkers were not tested against patients with other disease (for example, primary aldosteronism, thyroid disease). Future proof-of-concept studies are needed to confirm clinical usefulness of the proposed biomarkers as a noninvasive test for prognosis of HF development.

## Conclusions

This study demonstrated an altered gene expression profile in PBMCs during acute myocardial infarction and through the follow-up. We have identified a set of genes whose expression on the first day of AMI differed significantly between patients who developed HF during 6 months of observation and those who did not. The identified transcripts: *FMN1*, *JDP2*, and *RNASE1* may serve as novel prognostic biomarkers for the development of HF after AMI.

## Additional files

Additional file 1:
**Primer sequences and RT-qPCR conditions.**
Additional file 2:
**Baseline demographic and clinical characteristics of control groups.**
Additional file 3:
**Differentially expressed genes in patients on admission versus 6 months after AMI.**
Additional file 4:
**Differentially expressed genes common to admission versus 6 months after AMI and admission versus control.**
Additional file 5:
**Differentially expressed genes in patients on discharge versus 6 months after AMI.**
Additional file 6:
**Differentially expressed genes common to discharge versus 6 months after AMI and discharge versus control.**
Additional file 7:
**Differentially expressed genes in patients 1 month after AMI versus 6 months after MI.**
Additional file 8:
**Differentially expressed genes in HF patients versus non-HF patients.**
Additional file 9:
**Differentially expressed genes in HF patients versus control patients.**
Additional file 10:
**Differentially expressed genes in non-HF patients versus control patients.**
Additional file 11:
**Expression data from microarray experiments for** ***FMN1*** **,** ***JDP2*** **,** ***RNASE1*** **, and** ***TIMP1*** **at admission.** The y-axis represents log_2_ normalized intensity of gene expression and the x-axis represents analyzed groups. The line inside the box represents the median of the samples in a group. Dots represent relative expression levels in individual HF (red), non-HF (blue), and control group patients (green).

## Abbreviations

ACS: Acute coronary syndrome
AMI: Acute myocardial infarction
AQP9: Aquaporin 9
AUC: Area under the curve
BNP: B-type natriuretic peptide
CAD: Coronary artery disease
CHD: Coronary heart disease
CRP: C-reactive protein
CVD: Cardiovascular disease
EGR1: Early growth response 1
eRNA: Extracellular RNA
FAM20A: Family with sequence similarity 20, member A
FC: Fold change
FMN1: Formin 1
GEO: Gene expression omnibus
HF: Heart failure
IPA: Ingenuity pathway analysis
JDP2: Jun dimerization protein 2
LV: Left ventricular
LVEF: Left ventricular ejection fraction
NT-proBNP: N-terminal pro-brain natriuretic peptide
PBMCs: Peripheral blood mononuclear cells
PCI: Percutaneous coronary interventions
PPARG: Peroxisome proliferator-activated receptor gamma
RMA: Robust multi-array average
RNASE1: Ribonuclease, RNase A family, 1 (pancreatic)
ROC: Receiver operating characteristic
ROS: Reactive oxygen species
RT-qPCR: Real time reverse transcription-polymerase chain reaction
SOCS3: Suppressor of cytokine signaling 3
SRF: Serum response factor
STEMI: ST-segment elevation myocardial infarction
TIMP1: TIMP metallopeptidase inhibitor 1
