# Supplementary material for: Gene expression profiling reveals potential prognostic biomarkers associated with the progression of heart failure
Source: Genome Med. 2015 Mar 14;7(1):26. doi: 10.1186/s13073-015-0149-z (PMC4432772; doi:10.1186/s13073-015-0149-z)
Supplement: Additional file 1: — Primer sequences and RT-qPCR conditions. [file 13073_2015_149_MOESM1_ESM.doc]

**Additional file 1.** Primer sequences and RT-qPCR conditions

| **Gene symbol** | **Gene assignment** | **RefSeq** | **Sequence of primer** | **Product size (bp)** | **Efficiency** | **Annealing T (°C)** | **Extension time** | **Exon position** |
| --- | --- | --- | --- | --- | --- | --- | --- | --- |
| *AQP9* | Aquaporin 9 | AB008775 | F: 5'AGTGAGGACCACAACAGGTA3'  R: 5'AGCCACATCCAAGGACAATC3' | 186 | 0.78 | 50 | 5 | 1-2 |
| *FAM20A* | Family with sequence similarity 20, member A | BC036222 | F: 5'GGACCGGCACCATTATGAGA3'  R: 5'AGAGGCGAGAGGATGGAGAT3' | 120 | 0.99 | 56 | 5 | 9-10 |
| *FMN1* | Formin 1 | NM_001277313 | F: 5'CCAAGAGGATGAGCTGGTTA3'  R: 5'AGCTCGCGTGATGATCTCTA3' | 208 | 1.00 | 56 | 9 | 8-9 |
| *HPRT1* | Hypoxanthine phosphoribosyltransferase 1 | NM_000194 | F: 5'TGACCTTGATTTATTTTGCATACC3'  R: 5'CGAGCAAGACGTTCAGTCCT3' | 102 | 0.70 | 55 | 5 | 2-3 |
| *JDP2* | Jun dimerization protein 2 | NM_001135049 | F: 5'TACGCTGACATCCGCAACCT3'  R: 5'AACTCCGTGCGCTCCTTCTT3' | 188 | 1.00 | 58 | 7 | 2-3 |
| *PPARG* | Peroxisome proliferator-activated receptor gamma | NM_138712 | F: 5'ATGCTGGCCTCCTTGATGAA3'  R: 5'TGCCAAGTCGCTGTCATCTA3' | 171 | 0.92 | 58 | 7 | 7 |
| *RNASE1* | Ribonuclease, RNase A family, 1 (pancreatic) | NM_198232 | F: 5'GTCCGGCTCCTTCTGCTTGT3'  R: 5'GTGTCATATTCCGGCGCCTC3' | 172 | 1.00 | 58 | 9 | 1-2 |
| *SOCS3* | Suppressor of cytokine signaling 3 | BC060858 | F: 5'AGGCTCCTGGTAGAGAAGAC3'  R: 5'CCATCCAGGCTGAGTATGTG3' | 135 | 0.90 | 50 | 5 | 2 |
| *TIMP1* | TIMP metallopeptidase inhibitor 1 | NM_003254 | F: 5'GCTTCTGGCATCCTGTTGTT3'  R: 5'AGGTGGTCTGGTTGACTTCT3' | 148 | 0.72 | 54 | 6 | 2-3 |
