# Supplementary material for: Gene expression profiling reveals potential prognostic biomarkers associated with the progression of heart failure
Source: Genome Med. 2015 Mar 14;7(1):26. doi: 10.1186/s13073-015-0149-z (PMC4432772; doi:10.1186/s13073-015-0149-z)
Supplement: Additional file 2: — Baseline demographic and clinical characteristics of control groups. [file 13073_2015_149_MOESM2_ESM.doc]

**Additional file 2.** Baseline demographic and clinical characteristics of control groups

Data at admission

| **Characteristics** | **Control group I (*n* = 46)** | **Control group II (*n* = 21)** |
| --- | --- | --- |
| Men | 30 (65.2%) | 13 (61.9%) |
| Women | 16 (34.8%) | 8 (38.1%) |
| Age (years) | 58.0 ± 12.7 | 60.9 ± 9.7 |
| BMI (kg/m^2^) | 28.2 ± 5.4 | 28.8 ± 2.3 |
| Hypertension | 32 (69.6%) | 14 (66.7%) |
| Diabetes | 9 (19.6%) | 0 (0%) |
| Previous MI | 0 (0%) | 0 (0%) |
| Smoking | 17 (37.0%) | 3 (14.3%) |
| Hypercholesterolemia | 15 (32.6%) | 5 (23.8%) |
| Anterior myocardial infarction | 0 (0%) | 0 (0%) |
| Previous revascularization | 6 (13.0%) | no data |
| Noncoronary atherosclerosis | 7 (15.2%) | no data |
| NT-proBNP (pg/ml) | 91.3 ± 38.7 | no data |
| LVEF (%) | 54.8 ± 7.2 | no data |
| Medications | |  |
| Aspirin | 17 (36.9%) | 20 (95.2%) |
| Clopidogrel | 4 (8.7%) | 2 (9.5%) |
| Beta blockers | 41 (89.1%) | 20 (95.2%) |
| ACE inhibitors | 35 (76.1%) | 17 (80.9%) |
| Statins | 26 (56.5%) | 18 (85.7%) |
| Diuretics | 13 (28.3%) | no data |

Data is presented as mean value ± standard deviation or number or percentage of patients. BMI – Body Mass Index; MI – Myocardial Infarction; NT-proBNP – N-terminal pro-Brain Natriuretic Peptide; LVEF – Left Ventricular Ejection Fraction; ACE – Angiotensin-Converting Enzyme;
