# Supplementary material for: Gene expression profiling reveals potential prognostic biomarkers associated with the progression of heart failure
Source: Genome Med. 2015 Mar 14;7(1):26. doi: 10.1186/s13073-015-0149-z (PMC4432772; doi:10.1186/s13073-015-0149-z)
Supplement: Additional file 3: — Differentially expressed genes in patients on admission versus 6 months after AMI. [file 13073_2015_149_MOESM3_ESM.doc]

**Additional file 3. Differentially expressed genes in patients on admission versus 6 months after AMI**

| **Gene Symbol** | **RefSeq** | **Gene assignment** | ***p*-value** | **Fold Change** |
| --- | --- | --- | --- | --- |
| **SOCS3** | BC060858 | suppressor of cytokine signaling 3 | 1.02E-44 | 2.551 |
| **HP** | AK314700 | haptoglobin | 7.68E-18 | 2.093 |
| SLED1 | AY358224 | proteoglycan 3 pseudogene | 2.64E-26 | 2.045 |
| **FAM20A** | BC036222 | family with sequence similarity 20, member A | 5.36E-21 | 1.908 |
| ECRP | NR_033909 | ribonuclease, RNase A family, 2 (liver, eosinophil-derived neurotoxin) pseudogene | 1.73E-25 | 1.865 |
| **AQP9** | AB008775 | aquaporin 9 | 5.50E-28 | 1.862 |
| FCGR1B | BC110416 | Fc fragment of IgG, high affinity Ib, receptor (CD64) | 1.17E-19 | 1.810 |
| **FCGR1A** | BC032634 | Fc fragment of IgG, high affinity Ia, receptor (CD64) | 4.10E-20 | 1.771 |
| CD163 | BC051281 | CD163 molecule | 9.03E-28 | 1.735 |
| **EGR1** | M62829 | early growth response 1 | 4.41E-07 | 1.731 |
| TNFAIP6 | BC030205 | tumor necrosis factor, alpha-induced protein 6 | 2.62E-13 | 1.731 |
| **CYP1B1** | U03688 | cytochrome P450, family 1, subfamily B, polypeptide 1 | 2.24E-20 | 1.699 |
| **RNASE2** | M24157 | ribonuclease, RNase A family, 2 (liver, eosinophil-derived neurotoxin) | 1.78E-18 | 1.664 |
| **VSIG4** | AY358341 | V-set and immunoglobulin domain containing 4 | 4.21E-23 | 1.659 |
| ACSL1 | AK292798 | acyl-CoA synthetase long-chain family member 1 | 2.16E-28 | 1.648 |
| PTGS2 | NM_000963 | prostaglandin-endoperoxide synthase 2 (prostaglandin G/H synthase) | 1.56E-08 | 1.647 |
| **DYSF** | AF075575 | dysferlin, limb girdle muscular dystrophy 2B (autosomal recessive) | 9.86E-21 | 1.640 |
| IL1R2 | U74649 | interleukin 1 receptor, type II | 7.47E-14 | 1.635 |
| **FMN1** | NM_001277313 | formin 1 | 3.95E-16 | 1.619 |
| **PPARG** | NM_138712 | peroxisome proliferator-activated receptor gamma | 1.48E-28 | 1.613 |
| FOSB | BC036724 | FBJ murine osteosarcoma viral oncogene homolog B | 4.12E-07 | 1.611 |
| PDK4 | U54617 | pyruvate dehydrogenase kinase, isozyme 4 | 1.89E-15 | 1.610 |
| DSC2 | BC063291 | desmocollin 2 | 2.81E-16 | 1.608 |
| MERTK | U08023 | c-mer proto-oncogene tyrosine kinase | 2.65E-18 | 1.587 |
| FOS | NM_005252 | FBJ murine osteosarcoma viral oncogene homolog | 9.28E-13 | 1.580 |
| **CR1** | NM_000573 | complement component (3b/4b) receptor 1 (Knops blood group) | 3.41E-24 | 1.573 |
| AREG | BC009799 | amphiregulin | 2.02E-07 | 1.572 |
| **ST14** | BC030532 | suppression of tumorigenicity 14 (colon carcinoma) | 9.01E-31 | 1.570 |
| TMEM144 | BC054487 | transmembrane protein 144 | 2.04E-17 | 1.567 |
| MIR21 | AY699265 | microRNA 21 | 1.01E-15 | 1.565 |
| **STAB1** | AB052956 | stabilin 1 | 6.78E-28 | 1.560 |
| **EGR2** | BC035625 | early growth response 2 | 2.06E-06 | 1.559 |
| S100A9 | NM_002965 | S100 calcium binding protein A9 | 1.50E-28 | 1.556 |
| NRG1 | AF176921 | neuregulin 1 | 2.45E-10 | 1.553 |
| FAM198B | NM_001128424 | family with sequence similarity 198, member B | 1.69E-27 | 1.544 |
| **TCN2** | BC001176 | transcobalamin II | 1.69E-19 | 1.535 |
| CES1 | AB119995 | carboxylesterase 1 | 3.80E-10 | 1.534 |
| RNA5SP387 | ENST00000364226 | RNA, 5S ribosomal pseudogene 387 | 7.29E-18 | 1.525 |
| MS4A4A | NM_024021 | membrane-spanning 4-domains, subfamily A, member 4A | 7.38E-14 | 1.516 |
| S100A12 | BC070294 | S100 calcium binding protein A12 | 1.87E-21 | 1.515 |
| IL1B | BC008678 | interleukin 1, beta | 7.47E-09 | 1.511 |
| STEAP4 | NM_024636 | STEAP family member 4 | 6.88E-12 | 1.511 |
| **RNASE1** | NM_198232 | ribonuclease, RNase A family, 1 (pancreatic) | 4.24E-16 | 1.504 |
| FPR2 | AK290557 | formyl peptide receptor 2 | 1.10E-14 | 1.492 |
| **TMEM176A** | AF258340 | transmembrane protein 176A | 0.00230455 | 1.489 |
| TRIB1 | BC063292 | tribbles homolog 1 (Drosophila) | 6.43E-15 | 1.484 |
| C19orf59 | AF461155 | chromosome 19 open reading frame 59 | 6.92E-30 | 1.476 |
| TDRD9 | NM_153046 | tudor domain containing 9 | 9.50E-17 | 1.475 |
| **HBEGF** | BC033097 | heparin-binding EGF-like growth factor | 2.20E-10 | 1.475 |
| NAMPT | NM_005746 | nicotinamide phosphoribosyltransferase | 2.15E-13 | 1.471 |
| PLBD1 | BC063561 | phospholipase B domain containing 1 | 1.52E-21 | 1.466 |
| CCR1 | NM_001295 | chemokine (C-C motif) receptor 1 | 5.18E-18 | 1.463 |
| GCA | BC005214 | grancalcin, EF-hand calcium binding protein | 7.57E-21 | 1.460 |
| SIGLEC16 | ENST00000602139 | sialic acid binding Ig-like lectin 16 (gene/pseudogene) | 3.10E-18 | 1.460 |
| ADM | BC015961 | adrenomedullin | 1.89E-29 | 1.460 |
| NFIL3 | NM_005384 | nuclear factor, interleukin 3 regulated | 1.70E-14 | 1.456 |
| **ASGR2** | NM_001181 | asialoglycoprotein receptor 2 | 2.40E-20 | 1.454 |
| KCNJ15 | NM_002243 | potassium inwardly-rectifying channel, subfamily J, member 15 | 1.06E-15 | 1.449 |
| LILRA3 | AF014923 | leukocyte immunoglobulin-like receptor, subfamily A (without TM d | 1.46E-09 | 1.446 |
| C3AR1 | AK290242 | complement component 3a receptor 1 | 7.94E-11 | 1.436 |
| FLVCR2 | AY260577 | feline leukemia virus subgroup C cellular receptor family, member 2 | 3.14E-23 | 1.434 |
| LIN7A | AF087693 | lin-7 homolog A (*C. elegans*) | 1.12E-15 | 1.433 |
| FLT3 | BC036028 | fms-related tyrosine kinase 3 | 4.16E-12 | 1.429 |
| FAM151B | AY358256 | family with sequence similarity 151, member B | 3.18E-20 | 1.419 |
| FPR1 | ENST00000595042 | formyl peptide receptor 1 | 1.53E-17 | 1.419 |
| TREM1 | AF196329 | triggering receptor expressed on myeloid cells 1 | 3.82E-11 | 1.414 |
| CCR2 | NM_001123396 | chemokine (C-C motif) receptor 2 | 2.10E-18 | 1.412 |
| F5 | M14335 | coagulation factor V (proaccelerin, labile factor) | 6.09E-20 | 1.410 |
| LILRB4 | AK292082 | leukocyte immunoglobulin-like receptor, subfamily B (with TM and ITIM domains), member 4 | 5.50E-24 | 1.404 |
| SLC1A3 | BC037310 | solute carrier family 1 (glial high affinity glutamate transporter), member 3 | 1.99E-20 | 1.404 |
| ANKRD34B | NM_001004441 | ankyrin repeat domain 34B | 1.77E-15 | 1.401 |
| FFAR2 | AB378083 | free fatty acid receptor 2 | 3.51E-13 | 1.400 |
| CCR2 | NM_001123041 | chemokine (C-C motif) receptor 2 | 3.87E-17 | 1.400 |
| GPR34 | AK074627 | G protein-coupled receptor 34 | 8.21E-08 | 1.395 |
| C1QB | ENST00000509305 | complement component 1, q subcomponent, B chain | 2.57E-11 | 1.394 |
| MARC1 | NM_022746 | mitochondrial amidoxime reducing component 1 | 5.63E-15 | 1.394 |
| CYP27A1 | AK226039 | cytochrome P450, family 27, subfamily A, polypeptide 1 | 3.26E-12 | 1.393 |
| FOLR3 | NM_000804 | folate receptor 3 (gamma) | 0.000236351 | 1.392 |
| SERPINB2 | BC012609 | serpin peptidase inhibitor, clade B (ovalbumin), member 2 | 5.48E-09 | 1.387 |
| MAFB | BC036689 | v-maf musculoaponeurotic fibrosarcoma oncogene homolog B (avian) | 5.79E-18 | 1.385 |
| HLX | AB208812 | H2.0-like homeobox | 9.80E-22 | 1.382 |
| SH3PXD2B | NM_001017995 | SH3 and PX domains 2B | 8.50E-18 | 1.382 |
| LINC00189 | AF490769 | long intergenic non-protein coding RNA 189 | 2.45E-05 | 1.379 |
| EDA2R | AY152724 | ectodysplasin A2 receptor | 4.18E-40 | 1.376 |
| LILRA5 | AF499918 | leukocyte immunoglobulin-like receptor, subfamily A (with TM domain), member 5 | 3.31E-17 | 1.376 |
| EREG | BC136404 | epiregulin | 5.24E-09 | 1.375 |
| CD14 | BC010507 | CD14 molecule | 2.34E-19 | 1.371 |
| GLT1D1 | BC043528 | glycosyltransferase 1 domain containing 1 | 8.63E-22 | 1.370 |
| MIR24-2 | AF480559 | microRNA 24-2 | 1.46E-05 | 1.367 |
| VNN1 | U39664 | vanin 1 | 3.69E-07 | 1.366 |
| SLC26A8 | AF331522 | solute carrier family 26, member 8 | 1.18E-15 | 1.364 |
| IL8 | M17017 | interleukin 8 | 4.97E-05 | 1.364 |
| BCL6 | U00115 | B-cell CLL lymphoma 6 | 2.51E-18 | 1.364 |
| QPCT | BC047756 | glutaminyl-peptide cyclotransferase | 4.69E-15 | 1.363 |
| CLEC4E | AB024718 | C-type lectin domain family 4, member E | 2.57E-08 | 1.362 |
| MGST1 | J03746 | microsomal glutathione S-transferase 1 | 8.13E-14 | 1.360 |
| CD36 | L06850 | CD36 molecule (thrombospondin receptor) | 8.39E-20 | 1.360 |
| NR4A2 | BC009288 | nuclear receptor subfamily 4, group A, member 2 | 9.35E-05 | 1.359 |
| RBM47 | NM_001098634 | RNA binding motif protein 47 | 9.11E-23 | 1.359 |
| SERPINB10 | BC096217 | serpin peptidase inhibitor, clade B (ovalbumin), member 10 | 3.90E-06 | 1.357 |
| FAM20C | AF533706 | family with sequence similarity 20, member C | 5.19E-21 | 1.354 |
| C5AR1 | NM_001736 | complement component 5a receptor 1 | 1.69E-17 | 1.352 |
| NFE2 | AK290555 | nuclear factor (erythroid-derived 2), 45kDa | 1.89E-17 | 1.350 |
| MIR223 | NR_029637 | microRNA 223 | 3.96E-12 | 1.350 |
| MILR1 | NM_001085423 | mast cell immunoglobulin-like receptor 1 | 3.06E-16 | 1.349 |
| CR1L | BC109190 | complement component (3b/4b) receptor 1-like | 1.52E-17 | 1.349 |
| ADAM9 | AF495383 | ADAM metallopeptidase domain 9 | 4.41E-19 | 1.347 |
| PADI2 | NM_007365 | peptidyl arginine deiminase, type II | 2.06E-08 | 1.345 |
| MXD1 | BC098396 | MAX dimerization protein 1 | 1.02E-17 | 1.345 |
| BST1 | D21878 | bone marrow stromal cell antigen 1 | 4.13E-22 | 1.343 |
| THBD | NM_000361 | thrombomodulin | 3.66E-19 | 1.343 |
| GPER | NM_001039966 | G protein-coupled estrogen receptor 1 | 2.58E-22 | 1.343 |
| FCAR | U56236 | Fc fragment of IgA, receptor for | 4.45E-11 | 1.342 |
| LOC100652783 | AK311627 | putative POM121-like protein 1-like | 7.90E-09 | 1.340 |
| S100A8 | AK291328 | S100 calcium binding protein A8 | 5.83E-19 | 1.340 |
| IGJ | BC038982 | immunoglobulin J polypeptide, linker protein for immunoglobulin alpha | 0.00101084 | 1.338 |
| TLR2 | NM_003264 | toll-like receptor 2 | 5.44E-18 | 1.337 |
| BCAT1 | NM_005504 | branched chain amino-acid transaminase 1, cytosolic | 1.63E-13 | 1.336 |
| KLF4 | ENST00000493306 | Kruppel-like factor 4 (gut) | 1.30E-10 | 1.334 |
| DUSP1 | AK298047 | dual specificity phosphatase 1 | 1.59E-12 | 1.334 |
| MOP-1 | AB014771 | MOP-1 | 0.00194476 | 1.333 |
| MGAM | ENST00000475668 | maltase-glucoamylase (alpha-glucosidase) | 9.69E-07 | 1.332 |
| PYGL | BC009895 | phosphorylase, glycogen, liver | 4.80E-21 | 1.330 |
| ANPEP | BC058928 | alanyl (membrane) aminopeptidase | 1.10E-06 | 1.330 |
| RBP7 | AF399927 | retinol binding protein 7, cellular | 2.86E-14 | 1.330 |
| IL1RN | NM_173841 | interleukin 1 receptor antagonist | 9.88E-16 | 1.330 |
| TBC1D2 | AF318370 | TBC1 domain family, member 2 | 1.22E-25 | 1.329 |
| ZFP36 | BC009693 | ZFP36 ring finger protein | 5.78E-15 | 1.329 |
| LOC101060558 | ENST00000502466 | putative POM121-like protein 1-like | 1.96E-09 | 1.326 |
| RRP12 | BC012745 | ribosomal RNA processing 12 homolog (*S. cerevisiae*) | 2.79E-18 | 1.325 |
| ETS2 | NM_005239 | v-ets erythroblastosis virus E26 oncogene homolog 2 (avian) | 2.78E-18 | 1.324 |
| CEBPD | BC094715 | CCAAT enhancer binding protein (C/EBP), delta | 2.11E-22 | 1.323 |
| CREB5 | NM_182898 | cAMP responsive element binding protein 5 | 3.71E-20 | 1.322 |
| MRC1 | BC146838 | mannose receptor, C type 1 | 2.74E-12 | 1.322 |
| SLC11A1 | D38171 | solute carrier family 11 (proton-coupled divalent metal ion transporter), member 1 | 3.27E-16 | 1.320 |
| METTL7B | NM_152637 | methyltransferase like 7B | 9.97E-15 | 1.316 |
| CXCL16 | AF301016 | chemokine (C-X-C motif) ligand 16 | 4.39E-13 | 1.315 |
| VNN2 | AB026705 | vanin 2 | 3.86E-09 | 1.315 |
| LY96 | AB018549 | lymphocyte antigen 96 | 5.66E-11 | 1.315 |
| SMCO4 | AF197137 | single-pass membrane protein with coiled-coil domains 4 | 4.19E-13 | 1.315 |
| PGA4 | AK291864 | pepsinogen 4, group I (pepsinogen A) | 5.21E-06 | 1.311 |
| SASH1 | NM_015278 | SAM and SH3 domain containing 1 | 2.28E-09 | 1.310 |
| BCL2A1 | NM_001114735 | BCL2-related protein A1 | 4.92E-14 | 1.309 |
| CDA | BC048284 | cytidine deaminase | 2.54E-13 | 1.309 |
| CD93 | NM_012072 | CD93 molecule | 9.16E-21 | 1.309 |
| ADAP2 | BC033758 | ArfGAP with dual PH domains 2 | 5.70E-20 | 1.309 |
| PGA5 | BC029055 | pepsinogen 5, group I (pepsinogen A) | 5.10E-06 | 1.308 |
| ADAMTS2 | NM_014244 | ADAM metallopeptidase with thrombospondin type 1 motif, 2 | 2.52E-12 | 1.305 |
| LOC100653086 | ENST00000421606 | uncharacterized LOC100653086 | 2.31E-10 | 1.303 |
| LRP1 | NM_002332 | low density lipoprotein receptor-related protein 1 | 4.88E-22 | 1.302 |
| PGA3 | AK225679 | pepsinogen 3, group I (pepsinogen A) | 4.98E-06 | 1.302 |
| CSTA | NM_005213 | cystatin A (stefin A) | 2.58E-11 | 1.302 |
| CD180 | BC109069 | CD180 molecule | 6.18E-11 | 1.301 |
| GCNT4 | AF132035 | glucosaminyl (N-acetyl) transferase 4, core 2 | 6.22E-09 | -1.301 |
| NCR1 | ENST00000594765 | natural cytotoxicity triggering receptor 1 | 1.54E-12 | -1.303 |
| SNORD116-24 | NR_003338 | small nucleolar RNA, C D box 116-24 | 1.55E-07 | -1.305 |
| PRF1 | BC047695 | perforin 1 (pore forming protein) | 3.54E-11 | -1.306 |
| SLCO4C1 | AF119865 | solute carrier organic anion transporter family, member 4C1 | 3.56E-13 | -1.306 |
| ANKRD20A5P | ENST00000427989 | ankyrin repeat domain 20 family, member A5, pseudogene | 0.000178443 | -1.309 |
| PRSS23 | NM_007173 | protease, serine, 23 | 4.70E-11 | -1.316 |
| TFDP2 | U18422 | transcription factor Dp-2 (E2F dimerization partner 2) | 1.74E-18 | -1.319 |
| SNORD116-8 | NR_003323 | small nucleolar RNA, C D box 116-8 | 3.34E-06 | -1.319 |
| NELL2 | AK309825 | NEL-like 2 (chicken) | 1.82E-07 | -1.319 |
| C21orf15 | BX648100 | chromosome 21 open reading frame 15 | 0.00673521 | -1.322 |
| TRGV9 | ENST00000444775 | T cell receptor gamma variable 9 | 1.10E-06 | -1.323 |
| SNORD116-1 | NR_003316 | small nucleolar RNA, C D box 116-1 | 6.53E-06 | -1.326 |
| FAM169A | AK290004 | family with sequence similarity 169, member A | 1.97E-16 | -1.326 |
| AK5 | BC036666 | adenylate kinase 5 | 5.74E-07 | -1.328 |
| GNLY | BC063245 | granulysin | 7.39E-09 | -1.332 |
| ANKRD20A11P | AF426257 | ankyrin repeat domain 20 family, member A11, pseudogene | 0.000355049 | -1.336 |
| PDGFD | BC030645 | platelet derived growth factor D | 1.56E-10 | -1.337 |
| GPR56 | AY358400 | G protein-coupled receptor 56 | 2.93E-09 | -1.338 |
| ABCB1 | AK290159 | ATP-binding cassette, sub-family B (MDR TAP), member 1 | 1.51E-14 | -1.338 |
| KLRG1 | NM_005810 | killer cell lectin-like receptor subfamily G, member 1 | 9.38E-08 | -1.340 |
| CLC | NM_001828 | Charcot-Leyden crystal galectin | 0.00507986 | -1.342 |
| SAMD3 | NM_001017373 | sterile alpha motif domain containing 3 | 5.40E-14 | -1.343 |
| LINC00299 | AK127578 | long intergenic non-protein coding RNA 299 | 6.96E-07 | -1.344 |
| C1orf21 | AF312864 | chromosome 1 open reading frame 21 | 7.96E-13 | -1.346 |
| KLRC1 | NM_213658 | killer cell lectin-like receptor subfamily C, member 1 | 3.12E-07 | -1.346 |
| FGFBP2 | NM_031950 | fibroblast growth factor binding protein 2 | 7.78E-08 | -1.353 |
| HRH4 | AF312230 | histamine receptor H4 | 7.54E-07 | -1.357 |
| IL12RB2 | U64198 | interleukin 12 receptor, beta 2 | 3.32E-12 | -1.360 |
| IFI44L | ENST00000486882 | interferon-induced protein 44-like | 0.00196028 | -1.362 |
| TGFBR3 | NM_001195684 | transforming growth factor, beta receptor III | 5.34E-13 | -1.363 |
| KLRD1 | BC042884 | killer cell lectin-like receptor subfamily D, member 1 | 3.06E-11 | -1.364 |
| KLRF1 | AF175206 | killer cell lectin-like receptor subfamily F, member 1 | 4.03E-08 | -1.372 |
| GBP5 | AF430642 | guanylate binding protein 5 | 3.86E-13 | -1.373 |
| SH2D1B | AF484964 | SH2 domain containing 1B | 7.31E-09 | -1.375 |
| DTHD1 | NR_073198 | death domain containing 1 | 6.85E-10 | -1.387 |
| CEP78 | NM_032171 | centrosomal protein 78kDa | 8.48E-17 | -1.391 |
| GBP4 | AL832576 | guanylate binding protein 4 | 2.27E-16 | -1.415 |
| **KLRC2** | NM_002260 | killer cell lectin-like receptor subfamily C, member 2 | 7.14E-06 | -1.415 |
| ANKRD20A9P | ENST00000457997 | ankyrin repeat domain 20 family, member A9, pseudogene | 7.68E-06 | -1.433 |
| KLRC4-KLRK1 | AF461811 | KLRC4-KLRK1 readthrough | 5.06E-08 | -1.468 |
| **MYBL1** | BC101186 | v-myb myeloblastosis viral oncogene homolog (avian)-like 1 | 6.42E-20 | -1.480 |
| IL18RAP | NM_003853 | interleukin 18 receptor accessory protein | 1.95E-13 | -1.487 |
| **KLRC4** | AJ001683 | killer cell lectin-like receptor subfamily C, member 4 | 3.09E-11 | -1.490 |

The genes indicated in bold were identified in [21].
