# Supplementary material for: Gene expression profiling reveals potential prognostic biomarkers associated with the progression of heart failure
Source: Genome Med. 2015 Mar 14;7(1):26. doi: 10.1186/s13073-015-0149-z (PMC4432772; doi:10.1186/s13073-015-0149-z)
Supplement: Additional file 4: — Differentially expressed genes common to admission versus 6 months after AMI and admission versus control. [file 13073_2015_149_MOESM4_ESM.doc]

**Additional file 4.** Differentially expressed genes common to admission versus 6 months after AMI and admission versus control

|  | | | **admission versus 6 months** | | **admission versus control** | |
| --- | --- | --- | --- | --- | --- | --- |
| **Gene Symbol** | **RefSeq** | **Gene assignment** | ***p*-value** | **Fold change** | ***p*-value** | **Fold change** |
| SOCS3 | BC060858 | suppressor of cytokine signaling 3 | 1.0E-44 | 2.551 | 1.9E-07 | 1.840 |
| HP | AK314700 | haptoglobin | 7.7E-18 | 2.093 | 1.8E-04 | 1.832 |
| SLED1 | AY358224 | proteoglycan 3 pseudogene | 2.6E-26 | 2.045 | 1.8E-03 | 1.470 |
| FAM20A | BC036222 | family with sequence similarity 20, member A | 5.4E-21 | 1.908 | 3.3E-05 | 1.704 |
| ECRP | NR_033909 | ribonuclease, RNase A family, 2 (liver, eosinophil-derived neuroto | 1.7E-25 | 1.865 | 3.0E-07 | 1.765 |
| AQP9 | AB008775 | aquaporin 9 | 5.5E-28 | 1.862 | 3.4E-06 | 1.622 |
| FCGR1B | BC110416 | Fc fragment of IgG, high affinity Ib, receptor (CD64) | 1.2E-19 | 1.810 | 6.0E-03 | 1.398 |
| FCGR1A | BC032634 | Fc fragment of IgG, high affinity Ia, receptor (CD64) | 4.1E-20 | 1.771 | 6.6E-03 | 1.371 |
| CD163 | BC051281 | CD163 molecule | 9.0E-28 | 1.735 | 4.1E-07 | 1.602 |
| TNFAIP6 | BC030205 | tumor necrosis factor, alpha-induced protein 6 | 2.6E-13 | 1.731 | 2.6E-03 | 1.538 |
| CYP1B1 | U03688 | cytochrome P450, family 1, subfamily B, polypeptide 1 | 2.2E-20 | 1.699 | 5.6E-04 | 1.446 |
| RNASE2 | M24157 | ribonuclease, RNase A family, 2 (liver, eosinophil-derived neurotox | 1.8E-18 | 1.664 | 5.8E-05 | 1.552 |
| VSIG4 | AY358341 | V-set and immunoglobulin domain containing 4 | 4.2E-23 | 1.659 | 2.5E-06 | 1.566 |
| ACSL1 | AK292798 | acyl-CoA synthetase long-chain family member 1 | 2.2E-28 | 1.648 | 9.8E-05 | 1.380 |
| DYSF | AF075575 | dysferlin, limb girdle muscular dystrophy 2B (autosomal recessive) | 9.9E-21 | 1.640 | 1.5E-04 | 1.455 |
| IL1R2 | U74649 | interleukin 1 receptor, type II | 7.5E-14 | 1.635 | 3.3E-04 | 1.566 |
| FMN1 | NM_001277313 | formin 1 | 3.9E-16 | 1.619 | 5.3E-05 | 1.574 |
| PPARG | NM_138712 | peroxisome proliferator-activated receptor gamma | 1.5E-28 | 1.613 | 4.1E-07 | 1.494 |
| DSC2 | BC063291 | desmocollin 2 | 2.8E-16 | 1.608 | 5.7E-04 | 1.459 |
| MERTK | U08023 | c-mer proto-oncogene tyrosine kinase | 2.6E-18 | 1.587 | 1.2E-05 | 1.547 |
| CR1 | NM_000573 | complement component (3b/ 4b) receptor 1 (Knops blood group) | 3.4E-24 | 1.573 | 2.1E-08 | 1.596 |
| MIR21 | AY699265 | microRNA 21 | 1.0E-15 | 1.565 | 9.6E-03 | 1.313 |
| STAB1 | AB052956 | stabilin 1 | 6.8E-28 | 1.560 | 6.8E-08 | 1.499 |
| S100A9 | NM_002965 | S100 calcium binding protein A9 | 1.5E-28 | 1.556 | 9.7E-07 | 1.432 |
| NRG1 | AF176921 | neuregulin 1 | 2.4E-10 | 1.553 | 1.4E-05 | 1.793 |
| FAM198B | NM_001128424 | family with sequence similarity 198, member B | 1.7E-27 | 1.544 | 1.1E-04 | 1.326 |
| CES1 | AB119995 | carboxylesterase 1 | 3.8E-10 | 1.534 | 2.0E-03 | 1.499 |
| RNA5SP387 | ENST00000364226 | RNA, 5S ribosomal pseudogene 387 | 7.3E-18 | 1.525 | 1.2E-03 | 1.349 |
| MS4A4A | NM_024021 | membrane-spanning 4-domains, subfamily A, member 4A | 7.4E-14 | 1.516 | 3.1E-03 | 1.367 |
| S100A12 | BC070294 | S100 calcium binding protein A12 | 1.9E-21 | 1.515 | 2.3E-06 | 1.472 |
| STEAP4 | NM_024636 | STEAP family member 4 | 6.9E-12 | 1.511 | 6.6E-05 | 1.585 |
| RNASE1 | NM_198232 | ribonuclease, RNase A family, 1 (pancreatic) | 4.2E-16 | 1.504 | 1.0E-06 | 1.596 |
| FPR2 | AK290557 | formyl peptide receptor 2 | 1.1E-14 | 1.492 | 6.4E-03 | 1.306 |
| C19orf59 | AF461155 | chromosome 19 open reading frame 59 | 6.9E-30 | 1.476 | 2.4E-07 | 1.384 |
| TDRD9 | NM_153046 | tudor domain containing 9 | 9.5E-17 | 1.475 | 7.5E-05 | 1.419 |
| GCA | BC005214 | grancalcin, EF-hand calcium binding protein | 7.6E-21 | 1.460 | 2.0E-04 | 1.324 |
| ADM | BC015961 | adrenomedullin | 1.9E-29 | 1.460 | 1.8E-05 | 1.301 |
| ASGR2 | NM_001181 | asialoglycoprotein receptor 2 | 2.4E-20 | 1.454 | 4.0E-11 | 1.664 |
| KCNJ15 | NM_002243 | potassium inwardly-rectifying channel, subfamily J, member 15 | 1.1E-15 | 1.449 | 1.5E-04 | 1.395 |
| CCR2 | NM_001123396 | chemokine (C-C motif) receptor 2 | 2.1E-18 | 1.412 | 1.7E-04 | 1.322 |
| F5 | M14335 | coagulation factor V (proaccelerin, labile factor) | 6.1E-20 | 1.410 | 1.5E-05 | 1.357 |
| GPR34 | AK074627 | G protein-coupled receptor 34 | 8.2E-08 | 1.395 | 1.1E-02 | 1.355 |
| MARC1 | NM_022746 | mitochondrial amidoxime reducing component 1 | 5.6E-15 | 1.394 | 9.7E-04 | 1.304 |
| CYP27A1 | AK226039 | cytochrome P450, family 27, subfamily A, polypeptide 1 | 3.3E-12 | 1.393 | 5.4E-09 | 1.712 |
| FOLR3 | NM_000804 | folate receptor 3 (gamma) | 2.4E-04 | 1.392 | 3.7E-02 | 1.441 |
| EDA2R | AY152724 | ectodysplasin A2 receptor | 4.2E-40 | 1.376 | 7.3E-10 | 1.304 |
| CD14 | BC010507 | CD14 molecule | 2.3E-19 | 1.371 | 5.4E-06 | 1.350 |
| VNN1 | U39664 | vanin 1 | 3.7E-07 | 1.366 | 1.6E-02 | 1.331 |
| SLC26A8 | AF331522 | solute carrier family 26, member 8 | 1.2E-15 | 1.364 | 4.4E-06 | 1.403 |
| SERPINB10 | BC096217 | serpin peptidase inhibitor, clade B (ovalbumin), member 10 | 3.9E-06 | 1.357 | 9.0E-03 | 1.397 |
| MILR1 | NM_001085423 | mast cell immunoglobulin-like receptor 1 | 3.1E-16 | 1.349 | 6.7E-06 | 1.368 |
| S100A8 | AK291328 | S100 calcium binding protein A8 | 5.8E-19 | 1.340 | 4.7E-06 | 1.328 |
| VNN2 | AB026705 | vanin 2 | 3.9E-09 | 1.315 | 1.8E-03 | 1.322 |
| SASH1 | NM_015278 | SAM and SH3 domain containing 1 | 2.3E-09 | 1.310 | 3.6E-06 | 1.501 |
| BCL2A1 | NM_001114735 | BCL2-related protein A1 | 4.9E-14 | 1.309 | 1.1E-04 | 1.301 |
| LOC101060558 | ENST00000502466 | putative POM121-like protein 1-like | 1.9E-07 | 1.303 | 5.5E-03 | 1.313 |
| SLCO4C1 | AF119865 | solute carrier organic anion transporter family, member 4C1 | 3.6E-13 | -1.306 | 3.7E-05 | -1.336 |
| ANKRD20A5P | ENST00000427989 | ankyrin repeat domain 20 family, member A5, pseudogene | 1.8E-04 | -1.309 | 4.4E-02 | -1.324 |
| PRSS23 | NM_007173 | protease, serine, 23 | 4.7E-11 | -1.316 | 2.0E-05 | -1.410 |
| TRGV9 | ENST00000444775 | T cell receptor gamma variable 9 | 1.1E-06 | -1.323 | 2.1E-03 | -1.407 |
| GNLY | BC063245 | granulysin | 7.4E-09 | -1.332 | 4.6E-03 | -1.310 |
| PDGFD | BC030645 | platelet derived growth factor D | 1.6E-10 | -1.337 | 2.7E-04 | -1.374 |
| GPR56 | AY358400 | G protein-coupled receptor 56 | 2.9E-09 | -1.338 | 8.3E-05 | -1.452 |
| KLRG1 | NM_005810 | killer cell lectin-like receptor subfamily G, member 1 | 9.4E-08 | -1.340 | 2.6E-05 | -1.565 |
| CLC | NM_001828 | Charcot-Leyden crystal galectin | 5.1E-03 | -1.342 | 1.1E-02 | -1.682 |
| C1orf21 | AF312864 | chromosome 1 open reading frame 21 | 8.0E-13 | -1.346 | 8.4E-05 | -1.366 |
| KLRC1 | NM_213658 | killer cell lectin-like receptor subfamily C, member 1 | 3.1E-07 | -1.346 | 1.9E-02 | -1.302 |
| FGFBP2 | NM_031950 | fibroblast growth factor binding protein 2 | 7.8E-08 | -1.353 | 4.3E-03 | -1.363 |
| TGFBR3 | NM_001195684 | transforming growth factor, beta receptor III | 5.3E-13 | -1.363 | 3.7E-05 | -1.403 |
| KLRD1 | BC042884 | killer cell lectin-like receptor subfamily D, member 1 | 3.1E-11 | -1.364 | 3.7E-04 | -1.376 |
| GBP5 | AF430642 | guanylate binding protein 5 | 3.9E-13 | -1.373 | 1.1E-04 | -1.380 |
| CEP78 | NM_032171 | centrosomal protein 78kDa | 8.5E-17 | -1.391 | 1.8E-04 | -1.323 |
| GBP4 | AL832576 | guanylate binding protein 4 | 2.3E-16 | -1.415 | 1.6E-05 | -1.414 |
| KLRC2 | NM_002260 | killer cell lectin-like receptor subfamily C, member 2 | 7.1E-06 | -1.415 | 4.6E-02 | -1.348 |
| KLRC4-KLRK1 | AF461811 | KLRC4-KLRK1 readthrough | 5.1E-08 | -1.468 | 1.4E-02 | -1.396 |
| MYBL1 | BC101186 | v-myb myeloblastosis viral oncogene homolog (avian)-like 1 | 6.4E-20 | -1.480 | 1.4E-06 | -1.477 |
| KLRC4 | AJ001683 | killer cell lectin-like receptor subfamily C, member 4 | 3.1E-11 | -1.490 | 3.2E-04 | -1.513 |
