# Supplementary material for: Gene expression profiling reveals potential prognostic biomarkers associated with the progression of heart failure
Source: Genome Med. 2015 Mar 14;7(1):26. doi: 10.1186/s13073-015-0149-z (PMC4432772; doi:10.1186/s13073-015-0149-z)
Supplement: Additional file 5: — Differentially expressed genes in patients on discharge versus 6 months after AMI. [file 13073_2015_149_MOESM5_ESM.doc]

**Additional file 5.** Differentially expressed genes in patients on discharge versus 6 months after AMI

| **Gene Symbol** | **RefSeq** | **Gene assignment** | ***p*-value** | **Fold change** |
| --- | --- | --- | --- | --- |
| IGJ | BC038982 | immunoglobulin J polypeptide, linker protein for immunoglobulin alpha | 8.0E-10 | 1.748 |
| IGKV2D-29 | ENST00000491977 | immunoglobulin kappa variable 2D-29 | 1.0E-07 | 1.564 |
| IGKV3D-11 | ENST00000390277 | immunoglobulin kappa variable 3D-11 | 2.4E-09 | 1.552 |
| FAM20A | BC036222 | family with sequence similarity 20, member A | 8.7E-11 | 1.547 |
| IGKV1D-33 | ENST00000390265 | immunoglobulin kappa variable 1D-33 | 2.7E-09 | 1.529 |
| IGKV2-24 | ENST00000484817 | immunoglobulin kappa variable 2-24 | 5.0E-10 | 1.519 |
| IGKC | BC110394 | immunoglobulin kappa constant | 2.2E-10 | 1.518 |
| IGHG1 | BX640853 | immunoglobulin heavy constant gamma 1 (G1m marker) | 9.4E-10 | 1.508 |
| IGHV3-74 | ENST00000424969 | immunoglobulin heavy variable 3-74 | 1.5E-10 | 1.489 |
| IGHV3-72 | ENST00000433072 | immunoglobulin heavy variable 3-72 | 1.2E-10 | 1.476 |
| AC068279.2 | OTTHUMT00000330418 | *Homo sapiens* chromosome 2 clone RP11-153P14 | 9.1E-10 | 1.467 |
| IGKV1D-43 | ENST00000468879 | immunoglobulin kappa variable 1D-43 | 3.7E-09 | 1.465 |
| IGKV1D-16 | ENST00000492446 | immunoglobulin kappa variable 1D-16 | 3.6E-10 | 1.462 |
| IGHV3-20 | ENST00000390606 | immunoglobulin heavy variable 3-20 | 1.9E-09 | 1.457 |
| IGKV1D-27 | ENST00000453184 | immunoglobulin kappa variable 1D-27 (pseudogene) | 5.4E-06 | 1.433 |
| IGKV1D-12 | ENST00000390276 | immunoglobulin kappa variable 1D-12 | 1.5E-07 | 1.431 |
| IGHV3-48 | X81730 | immunoglobulin heavy variable 3-48 | 8.7E-10 | 1.427 |
| FCGR1B | BC110416 | Fc fragment of IgG, high affinity Ib, receptor (CD64) | 2.7E-08 | 1.426 |
| SOCS3 | BC060858 | suppressor of cytokine signaling 3 | 9.9E-09 | 1.415 |
| FCGR1A | BC032634 | Fc fragment of IgG, high affinity Ia, receptor (CD64) | 3.6E-08 | 1.397 |
| C1QB | ENST00000509305 | complement component 1, q subcomponent, B chain | 6.6E-11 | 1.387 |
| IGKV1OR10-1 | ENST00000442306 | immunoglobulin heavy variable 1 OR21-1 (pseudogene) | 3.4E-09 | 1.384 |
| IGKV2D-18 | ENST00000519785 | immunoglobulin kappa variable 2D-18 (pseudogene) | 1.8E-09 | 1.377 |
| TNFAIP6 | BC030205 | tumor necrosis factor, alpha-induced protein 6 | 1.5E-05 | 1.377 |
| AC127391.1 | OTTHUMT00000338373 | *Homo sapiens* chromosome UNK clone RP11-389I13 | 3.7E-09 | 1.369 |
| IGHV3-35 | ENST00000390617 | immunoglobulin heavy variable 3-35 (non-functional) | 1.0E-09 | 1.361 |
| TNFRSF17 | BC058291 | tumor necrosis factor receptor superfamily, member 17 | 1.3E-06 | 1.354 |
| GPR34 | AK074627 | G protein-coupled receptor 34 | 2.4E-06 | 1.341 |
| EPC1 | L23571 | enhancer of polycomb homolog 1 (*Drosophila*) | 1.3E-07 | 1.341 |
| IGHV3OR16-8 | ENST00000569103 | immunoglobulin heavy variable 3 OR16-8 (non-functional) | 4.5E-10 | 1.340 |
| IGHV4-59 | ENST00000390629 | immunoglobulin heavy variable 4-59 | 1.1E-05 | 1.338 |
| TMEM144 | BC054487 | transmembrane protein 144 | 4.3E-08 | 1.329 |
| NRG1 | AF176921 | neuregulin 1 | 5.4E-05 | 1.322 |
| IGLV6-57 | ENST00000390285 | immunoglobulin lambda variable 6-57 | 3.0E-08 | 1.320 |
| DSC2 | BC063291 | desmocollin 2 | 1.3E-06 | 1.317 |
| IGLV7-46 | ENST00000390295 | immunoglobulin lambda variable 7-46 (gene pseudogene) | 3.1E-06 | 1.316 |
| GPRC5D | AF209923 | G protein-coupled receptor, family C, group 5, member D | 6.7E-08 | 1.309 |
| ANPEP | BC058928 | alanyl (membrane) aminopeptidase | 4.9E-06 | 1.309 |
| HP | AK314700 | haptoglobin | 1.5E-03 | 1.302 |
| MS4A4A | NM_024021 | membrane-spanning 4-domains, subfamily A, member 4A | 1.7E-06 | 1.301 |
| C15orf54 | BX647708 | chromosome 15 open reading frame 54 | 2.2E-05 | -1.312 |
