# Supplementary material for: Gene expression profiling reveals potential prognostic biomarkers associated with the progression of heart failure
Source: Genome Med. 2015 Mar 14;7(1):26. doi: 10.1186/s13073-015-0149-z (PMC4432772; doi:10.1186/s13073-015-0149-z)
Supplement: Additional file 6: — Differentially expressed genes common to discharge versus 6 months after AMI and discharge versus control. [file 13073_2015_149_MOESM6_ESM.doc]

**Additional file 6.** Differentially expressed genes common to discharge versus 6 months after AMI and discharge versus control

|  | | | **discharge versus 6 months** | | **discharge versus control** | |
| --- | --- | --- | --- | --- | --- | --- |
| **Gene Symbol** | **RefSeq** | **Gene assignment** | ***p*-value** | **Fold change** | ***p*-value** | **Fold change** |
| IGJ | BC038982 | immunoglobulin J polypeptide, linker protein for immunoglobulin alpha | 8.0E-10 | 1.748 | 0.032 | 1.452 |
| IGKV2D-29 | ENST00000491977 | immunoglobulin kappa variable 2D-29 | 1.0E-07 | 1.564 | 0.014 | 1.492 |
| IGKV3D-11 | ENST00000390277 | immunoglobulin kappa variable 3D-11 | 2.4E-09 | 1.552 | 0.001 | 1.586 |
| FAM20A | BC036222 | family with sequence similarity 20, member A | 8.7E-11 | 1.547 | 0.012 | 1.382 |
| IGKV1D-33 | ENST00000390265 | immunoglobulin kappa variable 1D-33 | 2.7E-09 | 1.529 | 0.002 | 1.525 |
| IGKV2-24 | ENST00000484817 | immunoglobulin kappa variable 2-24 | 5.0E-10 | 1.519 | 0.009 | 1.402 |
| IGKC | BC110394 | immunoglobulin kappa constant | 2.2E-10 | 1.518 | 0.002 | 1.489 |
| IGHG1 | BX640853 | immunoglobulin heavy constant gamma 1 (G1m marker) | 9.4E-10 | 1.508 | 0.004 | 1.454 |
| IGHV3-74 | ENST00000424969 | immunoglobulin heavy variable 3-74 | 1.5E-10 | 1.489 | 0.002 | 1.441 |
| IGHV3-72 | ENST00000433072 | immunoglobulin heavy variable 3-72 | 1.2E-10 | 1.476 | 0.003 | 1.420 |
| AC068279.2 | OTTHUMT00000330418 | *Homo sapiens* chromosome 2 clone RP11-153P14 | 9.1E-10 | 1.467 | 0.003 | 1.425 |
| IGKV1D-43 | ENST00000468879 | immunoglobulin kappa variable 1D-43 | 3.7E-09 | 1.465 | 0.001 | 1.506 |
| IGKV1D-16 | ENST00000492446 | immunoglobulin kappa variable 1D-16 | 3.6E-10 | 1.462 | 0.004 | 1.394 |
| IGHV3-20 | ENST00000390606 | immunoglobulin heavy variable 3-20 | 1.9E-09 | 1.457 | 0.005 | 1.408 |
| IGKV1D-27 | ENST00000453184 | immunoglobulin kappa variable 1D-27 (pseudogene) | 5.4E-06 | 1.433 | 0.005 | 1.536 |
| IGKV1D-12 | ENST00000390276 | immunoglobulin kappa variable 1D-12 | 1.5E-07 | 1.431 | 0.007 | 1.429 |
| IGHV3-48 | X81730 | immunoglobulin heavy variable 3-48 | 8.7E-10 | 1.427 | 0.003 | 1.388 |
| IGKV1OR10-1 | ENST00000442306 | immunoglobulin heavy variable 1 | 3.4E-09 | 1.384 | 0.007 | 1.332 |
| AC127391.1 | OTTHUMT00000338373 | *Homo sapiens* chromosome UNK clone RP11-389I13 | 3.7E-09 | 1.369 | 0.008 | 1.311 |
| IGHV3-35 | ENST00000390617 | immunoglobulin heavy variable 3-35 (non-functional) | 1.0E-09 | 1.361 | 0.002 | 1.356 |
| GPR34 | AK074627 | G protein-coupled receptor 34 | 2.4E-06 | 1.341 | 0.029 | 1.303 |
| EPC1 | L23571 | enhancer of polycomb homolog 1 (*Drosophila*) | 1.3E-07 | 1.341 | 0.003 | 1.371 |
| IGHV3OR16-8 | ENST00000569103 | immunoglobulin heavy variable 3 | 4.5E-10 | 1.340 | 0.002 | 1.321 |
| IGHV4-59 | ENST00000390629 | immunoglobulin heavy variable 4-59 | 1.1E-05 | 1.338 | 0.001 | 1.522 |
| NRG1 | AF176921 | neuregulin 1 | 5.4E-05 | 1.322 | 0.002 | 1.525 |
| IGLV6-57 | ENST00000390285 | immunoglobulin lambda variable 6-57 | 3.0E-08 | 1.320 | 0.006 | 1.302 |
| C15orf54 | BX647708 | chromosome 15 open reading frame 54 | 2.2E-05 | -1.312 | 0.028 | -1.314 |
