# Supplementary material for: Gene expression profiling reveals potential prognostic biomarkers associated with the progression of heart failure
Source: Genome Med. 2015 Mar 14;7(1):26. doi: 10.1186/s13073-015-0149-z (PMC4432772; doi:10.1186/s13073-015-0149-z)
Supplement: Additional file 7: — Differentially expressed genes in patients 1 month after AMI versus 6 months after MI. [file 13073_2015_149_MOESM7_ESM.doc]

**Additional file 7.** Differentially expressed genes in patients 1 month after AMI versus 6 months after AMI

| **Gene Symbol** | **RefSeq** | **Gene assignment** | ***p*-value** | **Fold change** |
| --- | --- | --- | --- | --- |
| IGKV1D-33 | ENST00000390265 | immunoglobulin kappa variable 1D-33 | 5.4E-05 | 1.334 |
| IGJ | BC038982 | immunoglobulin J polypeptide, linker protein for immunoglobulin alpha | 7.4E-04 | 1.357 |
