# Supplementary material for: Gene expression profiling reveals potential prognostic biomarkers associated with the progression of heart failure
Source: Genome Med. 2015 Mar 14;7(1):26. doi: 10.1186/s13073-015-0149-z (PMC4432772; doi:10.1186/s13073-015-0149-z)
Supplement: Additional file 8: — Differentially expressed genes in HF patients versus non-HF patients. [file 13073_2015_149_MOESM8_ESM.doc]

**Additional file** **8.** Differentially expressed genes in HF patients versus non-HF patients

| **Gene Symbol** | **RefSeq** | **Gene assignment** | ***p*-value** | **Fold change** |
| --- | --- | --- | --- | --- |
| IL1R2 | BC039031 | interleukin 1 receptor, type II | 7.92E-03 | 3.167 |
| FMN1 | NM_001103184 | formin 1 | 1.06E-03 | 2.765 |
| VSIG4 | AY358341 | V-set and immunoglobulin domain containing 4 | 1.27E-02 | 2.380 |
| SLED1 | AY358224 | proteoglycan 3 pseudogene | 6.43E-03 | 2.302 |
| TNFAIP6 | BC030205 | tumor necrosis factor, alpha-induced protein 6 | 2.53E-02 | 2.160 |
| AREG | BC009799 | amphiregulin | 1.16E-02 | 2.090 |
| FLT3 | BC036028 | fms-related tyrosine kinase 3 | 8.35E-03 | 2.085 |
| RNASE1 | NM_198232 | ribonuclease, RNase A family, 1 (pancreatic) | 2.22E-03 | 2.044 |
| TPST1 | AF038009 | tyrosylprotein sulfotransferase 1 | 9.56E-03 | 2.011 |
| ADAMTS2 | ENST00000251582 | ADAM metallopeptidase with thrombospondin type 1 motif, 2 | 1.03E-02 | 1.960 |
| FOSB | BC036724 | FBJ murine osteosarcoma viral oncogene homolog B | 3.45E-02 | 1.954 |
| CLU | AY513288 | clusterin | 2.13E-02 | 1.938 |
| THBS1 | AK291639 | thrombospondin 1 | 4.82E-02 | 1.932 |
| TCN2 | BC001176 | transcobalamin II | 6.29E-03 | 1.880 |
| IL8 | M17017 | interleukin 8 | 4.53E-02 | 1.874 |
| MGAM | AF016833 | maltase-glucoamylase (alpha-glucosidase) | 1.05E-02 | 1.856 |
| PGA3 | AK225679 | pepsinogen 3, group I (pepsinogen A) | 7.42E-03 | 1.850 |
| JDP2 | NM_001135049 | Jun dimerization protein 2 | 1.13E-03 | 1.825 |
| PVALB | BC069300 | parvalbumin | 3.01E-02 | 1.770 |
| CD163 | Z22970 | CD163 molecule | 2.51E-02 | 1.768 |
| LYVE1 | AY358925 | lymphatic vessel endothelial hyaluronan receptor 1 | 6.53E-03 | 1.733 |
| MRC1 | J05550 | mannose receptor, C type 1 | 2.20E-02 | 1.723 |
| NRGN | U89165 | neurogranin (protein kinase C substrate, RC3) | 1.05E-02 | 1.720 |
| NFIL3 | U26173 | nuclear factor, interleukin 3 regulated | 7.78E-03 | 1.706 |
| NAMPT | AK292851 | nicotinamide phosphoribosyltransferase | 6.00E-03 | 1.703 |
| RN5S387 | ENST00000364226 | RNA, 5S ribosomal 387 | 1.47E-02 | 1.686 |
| G0S2 | BC009694 | G0 | 3.66E-03 | 1.686 |
| CLEC4E | AB024718 | C-type lectin domain family 4, member E | 4.54E-02 | 1.674 |
| FOS | BC004490 | FBJ murine osteosarcoma viral oncogene homolog | 1.48E-02 | 1.658 |
| C6orf25 | AJ292264 | chromosome 6 open reading frame 25 | 3.60E-02 | 1.653 |
| VNN1 | U39664 | vanin 1 | 3.76E-02 | 1.646 |
| FSTL1 | BC000055 | follistatin-like 1 | 2.73E-02 | 1.631 |
| PER1 | AF022991 | period homolog 1 (Drosophila) | 5.32E-03 | 1.624 |
| APBB2 | AK304247 | amyloid beta (A4) precursor protein-binding, family B, member 2 | 4.45E-03 | 1.615 |
| CDA | BC048284 | cytidine deaminase | 5.72E-03 | 1.606 |
| CLEC5A | AF139768 | C-type lectin domain family 5, member A | 2.88E-02 | 1.590 |
| TRIB1 | BC063292 | tribbles homolog 1 (Drosophila) | 2.19E-02 | 1.589 |
| SIGLEC16 | BC039008 | sialic acid binding Ig-like lectin 16 (gene | 3.76E-02 | 1.572 |
| LHFPL2 | AY309920 | lipoma HMGIC fusion partner-like 2 | 8.58E-03 | 1.562 |
| ST14 | AB030036 | suppression of tumorigenicity 14 (colon carcinoma) | 5.81E-03 | 1.560 |
| ADORA3 | AY358644 | adenosine A3 receptor | 3.47E-02 | 1.553 |
| GPER | AF015257 | G protein-coupled estrogen receptor 1 | 5.13E-03 | 1.529 |
| LY86 | BC038846 | lymphocyte antigen 86 | 2.43E-03 | 1.528 |
| ARRDC4 | BC028704 | arrestin domain containing 4 | 4.70E-03 | 1.527 |
| TIMP1 | NM_003254 | TIMP metallopeptidase inhibitor 1 | 2.49E-03 | 1.523 |
| MFSD2B | ENST00000406420 | major facilitator superfamily domain containing 2B | 2.51E-02 | 1.512 |
| ALOX15B | U78294 | arachidonate 15-lipoxygenase, type B | 4.06E-02 | 1.509 |
| SIRPB2 | AK294440 | signal-regulatory protein beta 2 | 6.02E-03 | 1.508 |
| CRISPLD2 | ENST00000262424 | cysteine-rich secretory protein LCCL domain containing 2 | 1.20E-02 | 1.503 |
| GCET2 | AY212246 | germinal center expressed transcript 2 | 4.27E-03 | -1.504 |
| CSGALNACT1 | AY358441 | chondroitin sulfate N-acetylgalactosaminyltransferase 1 | 4.69E-03 | -1.506 |
| SNORD42A | NR_000014 | small nucleolar RNA, C/D box 42A | 1.25E-02 | -1.510 |
| ZNF737 | ENST00000427401 | zinc finger protein 737 | 2.11E-02 | -1.510 |
| GYPE | BC017864 | glycophorin E (MNS blood group) | 2.10E-02 | -1.512 |
| TESPA1 | AB018291 | thymocyte expressed, positive selection associated 1 | 2.18E-03 | -1.516 |
| FAM153B | NM_001265615 | family with sequence similarity 153, member B | 9.65E-03 | -1.530 |
| TRIB2 | D87119 | tribbles homolog 2 (Drosophila) | 2.16E-03 | -1.530 |
| TAS2R4 | BC130439 | taste receptor, type 2, member 4 | 2.97E-03 | -1.530 |
| RHOH | BC014261 | ras homolog family member H | 1.25E-02 | -1.530 |
| MCM8 | AY158211 | minichromosome maintenance complex component 8 | 1.06E-04 | -1.530 |
| RASGRP3 | AK292983 | RAS guanyl releasing protein 3 (calcium and DAG-regulated) | 9.37E-03 | -1.531 |
| SNORD116-8 | NR_003323 | small nucleolar RNA, C/D box 116-8 | 4.82E-02 | -1.531 |
| CCR4 | BC071751 | chemokine (C-C motif) receptor 4 | 2.81E-02 | -1.535 |
| CR2 | AK301496 | complement component (3d/Epstein Barr virus) receptor 2 | 4.41E-03 | -1.537 |
| ZNF253 | BC065572 | zinc finger protein 253 | 5.73E-03 | -1.537 |
| ZNF493 | ENST00000392288 | zinc finger protein 493 | 2.63E-03 | -1.537 |
| IL2RA | K03122 | interleukin 2 receptor, alpha | 1.17E-02 | -1.538 |
| RPL13A | AK056837 | ribosomal protein L13a | 3.89E-02 | -1.540 |
| RALGPS2 | BC047391 | Ral GEF with PH domain and SH3 binding motif 2 | 1.41E-02 | -1.542 |
| P2RY10 | BC051875 | purinergic receptor P2Y, G-protein coupled, 10 | 6.18E-03 | -1.542 |
| SNORD116-3 | NR_003318 | small nucleolar RNA, C/D box 116-3 | 4.63E-02 | -1.543 |
| BANK1 | ENST00000504592 | B-cell scaffold protein with ankyrin repeats 1 | 1.59E-02 | -1.547 |
| HIVEP2 | DQ231041 | human immunodeficiency virus type I enhancer binding protein 2 | 6.68E-04 | -1.547 |
| ANK3 | U13616 | ankyrin 3, node of Ranvier (ankyrin G) | 2.11E-02 | -1.551 |
| COBLL1 | AK294937 | COBL-like 1 | 3.99E-03 | -1.553 |
| DPP4 | AB451339 | dipeptidyl-peptidase 4 | 1.60E-02 | -1.555 |
| RIC3 | BC022455 | resistance to inhibitors of cholinesterase 3 homolog (C. elegans) | 6.79E-03 | -1.557 |
| GPR174 | BC104922 | G protein-coupled receptor 174 | 1.70E-02 | -1.557 |
| GCNT4 | AF132035 | glucosaminyl (N-acetyl) transferase 4, core 2 | 4.38E-02 | -1.558 |
| RBMX | ENST00000565907 | RNA binding motif protein, X-linked | 2.39E-02 | -1.558 |
| EPHX2 | BC011628 | epoxide hydrolase 2, cytoplasmic | 4.21E-03 | -1.560 |
| DOCK9 | AB028981 | dedicator of cytokinesis 9 | 6.61E-03 | -1.562 |
| SNHG1 | AK095849 | small nucleolar RNA host gene 1 (non-protein coding) | 3.24E-02 | -1.563 |
| INPP4B | BC110918 | inositol polyphosphate-4-phosphatase, type II, 105kDa | 1.01E-02 | -1.567 |
| SNORD116-1 | NR_003316 | small nucleolar RNA, C | 4.32E-02 | -1.567 |
| MAL | BC003006 | mal, T-cell differentiation protein | 8.56E-03 | -1.571 |
| VSIG1 | BC043216 | V-set and immunoglobulin domain containing 1 | 7.48E-03 | -1.571 |
| SNHG12 | AK092096 | small nucleolar RNA host gene 12 (non-protein coding) | 8.99E-03 | -1.572 |
| GPR171 | BC036815 | G protein-coupled receptor 171 | 1.93E-02 | -1.573 |
| TMEM194B | AK296987 | transmembrane protein 194B | 7.18E-03 | -1.588 |
| IKZF2 | AF130863 | IKAROS family zinc finger 2 (Helios) | 1.62E-02 | -1.596 |
| OSBPL10 | AF392451 | oxysterol binding protein-like 10 | 5.15E-03 | -1.596 |
| ZNF860 | AK294003 | zinc finger protein 860 | 9.97E-03 | -1.604 |
| GAS5 | BC038733 | growth arrest-specific 5 (non-protein coding) | 1.25E-02 | -1.605 |
| AMY2B | BC011179 | amylase, alpha 2B (pancreatic) | 1.39E-03 | -1.606 |
| NMRK1 | AK023413 | nicotinamide riboside kinase 1 | 7.86E-03 | -1.606 |
| ANKRD36B | ENST00000443455 | ankyrin repeat domain 36B | 5.54E-03 | -1.610 |
| ICOS | BC028210 | inducible T-cell co-stimulator | 7.73E-03 | -1.616 |
| TRAT1 | AK291991 | T cell receptor associated transmembrane adaptor 1 | 1.74E-02 | -1.625 |
| FAM153A | AB018295 | family with sequence similarity 153, member A | 4.54E-03 | -1.625 |
| SNORD116-14 | NR_003329 | small nucleolar RNA, C | 2.93E-02 | -1.633 |
| ITGA6 | AK294436 | integrin, alpha 6 | 6.13E-03 | -1.638 |
| SNORA22 | NR_002961 | small nucleolar RNA, H | 7.12E-03 | -1.653 |
| FCRL1 | AF459634 | Fc receptor-like 1 | 1.08E-02 | -1.655 |
| CD28 | J02988 | CD28 molecule | 6.67E-03 | -1.655 |
| SNORD28 | NR_002562 | small nucleolar RNA, C/D box 28 | 1.34E-02 | -1.657 |
| MS4A3 | L35848 | membrane-spanning 4-domains, subfamily A, member 3 (hematopoietic cell-specific) | 3.84E-02 | -1.657 |
| HRH4 | AF312230 | histamine receptor H4 | 3.09E-02 | -1.664 |
| OR52N4 | BC137035 | olfactory receptor, family 52, subfamily N, member 4 | 8.01E-03 | -1.676 |
| ABCD2 | BC104901 | ATP-binding cassette, sub-family D (ALD), member 2 | 1.28E-02 | -1.691 |
| ZNF204P | AK296042 | zinc finger protein 204, pseudogene | 1.73E-02 | -1.719 |
| DSC1 | X72925 | desmocollin 1 | 4.64E-03 | -1.731 |
| SNORD116-24 | NR_003338 | small nucleolar RNA, C/D box 116-24 | 1.18E-02 | -1.742 |
| FCRL2 | AL833194 | Fc receptor-like 2 | 3.01E-03 | -1.745 |
| GSDMB | AF258572 | gasdermin B | 1.02E-03 | -1.750 |
| FADS2 | AF108658 | fatty acid desaturase 2 | 4.55E-02 | -1.750 |
| STAP1 | AB023483 | signal transducing adaptor family member 1 | 4.97E-03 | -1.751 |
| NELL2 | AK295125 | NEL-like 2 (chicken) | 2.31E-03 | -1.794 |
| MS4A1 | AK292168 | membrane-spanning 4-domains, subfamily A, member 1 | 1.06E-02 | -1.808 |
| TSHZ2 | AY926481 | teashirt zinc finger homeobox 2 | 1.47E-02 | -1.833 |
| CD24 | NM_013230 | CD24 molecule | 8.88E-03 | -1.863 |
| KLRC4-KLRK1 | AF461811 | KLRC4-KLRK1 readthrough | 4.70E-02 | -1.864 |
| ZNF429 | AY269786 | zinc finger protein 429 | 5.48E-05 | -1.902 |
| AK5 | AY171600 | adenylate kinase 5 | 5.74E-03 | -1.945 |
| LRRN3 | BC035133 | leucine rich repeat neuronal 3 | 6.03E-03 | -2.672 |
| GPR15 | BC101779 | G protein-coupled receptor 15 | 9.74E-03 | -3.138 |
