# Supplementary material for: Gene expression profiling reveals potential prognostic biomarkers associated with the progression of heart failure
Source: Genome Med. 2015 Mar 14;7(1):26. doi: 10.1186/s13073-015-0149-z (PMC4432772; doi:10.1186/s13073-015-0149-z)
Supplement: Additional file 9: — Differentially expressed genes in HF patients versus control patients. [file 13073_2015_149_MOESM9_ESM.doc]

**Additional file** **9.** Differentially expressed genes in HF patients versus control patients

| **Gene Symbol** | **RefSeq** | **Gene assignment** | ***p*-value** | **Fold change** |
| --- | --- | --- | --- | --- |
| TMEM176A | AF258340 | transmembrane protein 176A | 1.16E-02 | 3.876 |
| ECRP | NR_033909 | ribonuclease, RNase A family, 2 (liver, eosinophil-derived neurotoxin) pseudogene | 5.57E-04 | 3.196 |
| FMN1 | NM_001103184 | formin 1 | 4.31E-04 | 3.081 |
| IL1R2 | BC039031 | interleukin 1 receptor, type II | 9.50E-03 | 2.947 |
| VSIG4 | AY358341 | V-set and immunoglobulin domain containing 4 | 5.46E-03 | 2.679 |
| HP | L29394 | haptoglobin | 5.64E-03 | 2.614 |
| RNASE1 | NM_198232 | ribonuclease, RNase A family, 1 (pancreatic) | 2.44E-04 | 2.612 |
| TCN2 | BC001176 | transcobalamin II | 3.74E-04 | 2.593 |
| RNASE2 | M28129 | ribonuclease, RNase A family, 2 (liver, eosinophil-derived neurotoxin) | 3.27E-03 | 2.504 |
| AQP9 | AF016495 | aquaporin 9 | 5.54E-04 | 2.468 |
| CD163 | Z22970 | CD163 molecule | 2.47E-03 | 2.355 |
| SOCS3 | BC060858 | suppressor of cytokine signaling 3 | 6.21E-03 | 2.340 |
| STAB1 | AB052956 | stabilin 1 | 2.47E-04 | 2.324 |
| SIGLEC16 | BC039008 | sialic acid binding Ig-like lectin 16 (gene/pseudogene) | 2.32E-03 | 2.320 |
| C1QC | AF087892 | complement component 1, q subcomponent, C chain | 2.78E-03 | 2.269 |
| PPARG | ENST00000397010 | peroxisome proliferator-activated receptor gamma | 1.96E-03 | 2.258 |
| LINC00189 | AF490769 | long intergenic non-protein coding RNA 189 | 3.23E-02 | 2.255 |
| C1QB | BC008983 | complement component 1, q subcomponent, B chain | 4.73E-03 | 2.224 |
| SLED1 | AY358224 | proteoglycan 3 pseudogene | 1.20E-02 | 2.217 |
| ADAMTS2 | ENST00000251582 | ADAM metallopeptidase with thrombospondin type 1 motif, 2 | 3.67E-03 | 2.193 |
| FAM20A | BC036222 | family with sequence similarity 20, member A | 2.79E-02 | 2.158 |
| MSR1 | BC063878 | macrophage scavenger receptor 1 | 2.66E-02 | 2.140 |
| MERTK | BC114918 | c-mer proto-oncogene tyrosine kinase | 3.03E-03 | 2.136 |
| TNFAIP6 | BC030205 | tumor necrosis factor, alpha-induced protein 6 | 3.32E-02 | 2.134 |
| PADI2 | AB030176 | peptidyl arginine deiminase, type II | 2.20E-03 | 2.090 |
| MS4A4A | AF237912 | membrane-spanning 4-domains, subfamily A, member 4A | 1.24E-02 | 2.072 |
| FLT3 | BC036028 | fms-related tyrosine kinase 3 | 7.89E-03 | 2.055 |
| GPR34 | AK074627 | G protein-coupled receptor 34 | 2.34E-02 | 2.026 |
| ST14 | AB030036 | suppression of tumorigenicity 14 (colon carcinoma) | 3.53E-04 | 2.017 |
| MRC1 | J05550 | mannose receptor, C type 1 | 5.38E-03 | 2.005 |
| CR1 | ENST00000367049 | complement component (3b/4b) receptor 1 | 2.75E-03 | 1.973 |
| SH3PXD2B | ENST00000311601 | SH3 and PX domains 2B | 5.44E-03 | 1.960 |
| SERPINB10 | BC096217 | serpin peptidase inhibitor, clade B (ovalbumin), member 10 | 8.35E-03 | 1.955 |
| TPST1 | AF038009 | tyrosylprotein sulfotransferase 1 | 1.02E-02 | 1.954 |
| SLC1A3 | BC037310 | solute carrier family 1 (glial high affinity glutamate transporter), member 3 | 6.04E-03 | 1.940 |
| LYVE1 | AY358925 | lymphatic vessel endothelial hyaluronan receptor 1 | 2.01E-03 | 1.918 |
| RN5S387 | ENST00000364226 | RNA, 5S ribosomal 387 | 7.18E-03 | 1.891 |
| ASGR2 | AF529374 | asialoglycoprotein receptor 2 | 1.19E-03 | 1.889 |
| KCTD12 | AF359381 | potassium channel tetramerisation domain containing 12 | 2.07E-04 | 1.882 |
| DYSF | ENST00000409366 | dysferlin | 4.28E-03 | 1.880 |
| SASH1 | ENST00000367467 | SAM and SH3 domain containing 1 | 1.83E-03 | 1.871 |
| FAM198B | BC043193 | family with sequence similarity 198, member B | 6.19E-04 | 1.869 |
| FCGR1A | AK291502 | Fc fragment of IgG, high affinity Ia, receptor (CD64) | 4.82E-03 | 1.859 |
| DSC2 | BC063291 | desmocollin 2 | 5.94E-03 | 1.839 |
| SLC11A1 | AK303398 | solute carrier family 11 (proton-coupled divalent metal ion transporter), member 1 | 3.91E-04 | 1.825 |
| SRGAP1 | BC053903 | SLIT-ROBO Rho GTPase activating protein 1 | 2.92E-02 | 1.817 |
| LOC728093 | AK296222 | putative POM121-like protein 1-like | 1.40E-02 | 1.813 |
| GFRA2 | BC041688 | GDNF family receptor alpha 2 | 6.76E-03 | 1.802 |
| TRIB1 | BC063292 | tribbles homolog 1 (*Drosophila*) | 5.54E-03 | 1.802 |
| FLVCR2 | AY260577 | feline leukemia virus subgroup C cellular receptor family, member | 1.03E-03 | 1.797 |
| MGST1 | J03746 | microsomal glutathione S-transferase 1 | 1.94E-02 | 1.797 |
| MIR223 | NR_029637 | microRNA 223 | 1.54E-02 | 1.788 |
| CYP1B1 | U03688 | cytochrome P450, family 1, subfamily B, polypeptide 1 | 4.72E-03 | 1.786 |
| CTSD | BC016320 | cathepsin D | 1.61E-04 | 1.781 |
| FPR2 | BC071722 | formyl peptide receptor 2 | 3.51E-03 | 1.768 |
| ADM | BC015961 | adrenomedullin | 6.10E-04 | 1.767 |
| RASGRP4 | AY048120 | RAS guanyl releasing protein 4 | 8.02E-04 | 1.766 |
| LGALS9 | AK126017 | lectin, galactoside-binding, soluble, 9 | 4.25E-03 | 1.753 |
| LIN7A | AF173081 | lin-7 homolog A (*C. elegans*) | 5.47E-03 | 1.742 |
| ADORA3 | AY358644 | adenosine A3 receptor | 1.04E-02 | 1.740 |
| FPR3 | BC059388 | formyl peptide receptor 3 | 9.63E-03 | 1.740 |
| KIAA1598 | AK091578 | KIAA1598 | 1.97E-03 | 1.739 |
| SIGLEC9 | BC035365 | sialic acid binding Ig-like lectin 9 | 2.18E-03 | 1.734 |
| MAFB | BC036689 | v-maf musculoaponeurotic fibrosarcoma oncogene homolog B (avian) | 7.52E-04 | 1.731 |
| HSPA1A | BC018740 | heat shock 70kDa protein 1A | 1.33E-03 | 1.722 |
| HSPA1B | BC063507 | heat shock 70kDa protein 1B | 1.33E-03 | 1.722 |
| CCR1 | L10918 | chemokine (C-C motif) receptor 1 | 4.26E-03 | 1.721 |
| LILRA2 | AF025531 | leukocyte immunoglobulin-like receptor, subfamily A (with TM domain), member 2 | 2.53E-04 | 1.716 |
| ACSL1 | AK292798 | acyl-CoA synthetase long-chain family member 1 | 6.85E-03 | 1.708 |
| GPER | AF015257 | G protein-coupled estrogen receptor 1 | 1.04E-03 | 1.704 |
| CR1L | BC109190 | complement component (3b/4b) receptor 1-like | 3.53E-03 | 1.703 |
| METTL7B | AY358508 | methyltransferase like 7B | 1.43E-02 | 1.700 |
| CPNE2 | AF492484 | copine II | 8.21E-04 | 1.699 |
| QSOX1 | U97276 | quiescin Q6 sulfhydryl oxidase 1 | 3.35E-04 | 1.690 |
| CLEC10A | BC027858 | C-type lectin domain family 10, member A | 2.81E-04 | 1.688 |
| CDA | BC048284 | cytidine deaminase | 2.77E-03 | 1.682 |
| KCNJ15 | BC013327 | potassium inwardly-rectifying channel, subfamily J, member 15 | 3.65E-03 | 1.682 |
| FCGR2A | BC020823 | Fc fragment of IgG, low affinity IIa, receptor (CD32) | 5.41E-03 | 1.668 |
| THBD | BC035602 | thrombomodulin | 4.03E-03 | 1.657 |
| CRISPLD2 | ENST00000262424 | cysteine-rich secretory protein LCCL domain containing 2 | 3.08E-03 | 1.657 |
| TDRD9 | BC128057 | tudor domain containing 9 | 1.58E-03 | 1.657 |
| LDLRAD3 | BC042754 | low density lipoprotein receptor class A domain containing 3 | 1.80E-03 | 1.654 |
| OLFML2B | BC067274 | olfactomedin-like 2B | 9.52E-04 | 1.652 |
| FAM151B | AY358256 | family with sequence similarity 151, member B | 1.73E-03 | 1.650 |
| SIGLEC7 | BC028150 | sialic acid binding Ig-like lectin 7 | 4.68E-04 | 1.649 |
| TMEM150B | BC171904 | transmembrane protein 150B | 1.15E-03 | 1.648 |
| RGL1 | AF186780 | ral guanine nucleotide dissociation stimulator-like 1 | 1.29E-02 | 1.648 |
| S100A12 | D83664 | S100 calcium binding protein A12 | 2.12E-02 | 1.646 |
| GM2A | BC009273 | GM2 ganglioside activator | 3.07E-03 | 1.640 |
| MGAM | AF016833 | maltase-glucoamylase (alpha-glucosidase) | 2.64E-02 | 1.639 |
| PDK4 | BC040239 | pyruvate dehydrogenase kinase, isozyme 4 | 4.32E-02 | 1.639 |
| F5 | M16967 | coagulation factor V (proaccelerin, labile factor) | 4.39E-03 | 1.637 |
| PLA2G15 | AB017494 | phospholipase A2, group XV | 1.43E-04 | 1.637 |
| LILRB4 | AK292082 | leukocyte immunoglobulin-like receptor, subfamily B (with TM and ITIM domains), member 4 | 9.64E-04 | 1.635 |
| IL1RN | BC068441 | interleukin 1 receptor antagonist | 2.73E-03 | 1.628 |
| TMEM106A | BC012139 | transmembrane protein 106A | 8.70E-04 | 1.623 |
| C3AR1 | U62027 | complement component 3a receptor 1 | 2.58E-02 | 1.620 |
| CSF3R | BC053585 | colony stimulating factor 3 receptor (granulocyte) | 2.12E-03 | 1.619 |
| RNASE3 | M28128 | ribonuclease, RNase A family, 3 | 1.17E-02 | 1.615 |
| TBC1D2 | AY026527 | TBC1 domain family, member 2 | 1.11E-03 | 1.610 |
| RBM47 | AF262323 | RNA binding motif protein 47 | 7.16E-04 | 1.609 |
| TPP1 | AY268890 | tripeptidyl peptidase I | 2.45E-04 | 1.608 |
| ST6GALNAC3 | BC059363 | ST6 (alpha-N-acetyl-neuraminyl-2,3-beta-galactosyl-1,3)-N-acetylgalactosaminide alpha-2,6-sialyltransferase 3 | 4.12E-03 | 1.606 |
| ALOX15B | U78294 | arachidonate 15-lipoxygenase, type B | 2.02E-02 | 1.601 |
| JDP2 | NM_001135049 | Jun dimerization protein 2 | 6.25E-03 | 1.597 |
| CREG1 | BC008628 | cellular repressor of E1A-stimulated genes 1 | 2.04E-03 | 1.596 |
| ADAP2 | BC033758 | ArfGAP with dual PH domains 2 | 9.77E-04 | 1.593 |
| VDR | AF026260 | vitamin D (1,25- dihydroxyvitamin D3) receptor | 1.69E-03 | 1.588 |
| MILR1 | ENST00000571603 | mast cell immunoglobulin-like receptor 1 | 3.56E-03 | 1.586 |
| HRH2 | BC054510 | histamine receptor H2 | 6.28E-03 | 1.586 |
| FBN2 | ENST00000508053 | fibrillin 2 | 1.50E-02 | 1.579 |
| BCL6 | AK304325 | B-cell CLL | 3.37E-03 | 1.576 |
| CD300LB | BC028091 | CD300 molecule-like family member b | 1.41E-03 | 1.573 |
| PLB1 | BC150633 | phospholipase B1 | 2.41E-03 | 1.570 |
| SLC36A1 | BX537963 | solute carrier family 36 (proton/amino acid symporter), member 1 | 1.92E-03 | 1.567 |
| SIRPB2 | AK294440 | signal-regulatory protein beta 2 | 3.07E-03 | 1.564 |
| PHC2 | AK292905 | polyhomeotic homolog 2 (*Drosophila*) | 6.32E-04 | 1.562 |
| DACH1 | AF102546 | dachshund homolog 1 (*Drosophila*) | 2.08E-03 | 1.559 |
| C1QA | BC030153 | complement component 1, q subcomponent, A chain | 1.81E-02 | 1.557 |
| FGD2 | AK092732 | FYVE, RhoGEF and PH domain containing 2 | 1.86E-03 | 1.557 |
| ATP6V0A1 | BC032398 | ATPase, H+ transporting, lysosomal V0 subunit a1 | 1.12E-03 | 1.557 |
| CTTNBP2NL | BC016029 | CTTNBP2 N-terminal like | 1.15E-03 | 1.555 |
| HLX | M60721 | H2.0-like homeobox | 3.93E-03 | 1.553 |
| GPR84 | BC020614 | G protein-coupled receptor 84 | 1.13E-02 | 1.548 |
| LILRA5 | AF324830 | leukocyte immunoglobulin-like receptor, subfamily A (with TM domain), member 5 | 8.50E-03 | 1.548 |
| NFE2 | BC005044 | nuclear factor (erythroid-derived 2), 45kDa | 1.58E-02 | 1.548 |
| ZNF467 | BC052625 | zinc finger protein 467 | 1.40E-03 | 1.543 |
| PGA3 | AK225679 | pepsinogen 3, group I (pepsinogen A) | 3.49E-02 | 1.538 |
| MARC1 | BC010619 | mitochondrial amidoxime reducing component 1 | 2.21E-02 | 1.538 |
| CD33 | BC028152 | CD33 molecule | 1.82E-03 | 1.534 |
| DLG4 | AK293835 | discs, large homolog 4 (*Drosophila*) | 4.66E-03 | 1.532 |
| C19orf59 | AF461155 | chromosome 19 open reading frame 59 | 4.57E-03 | 1.532 |
| CA5BP1 | ENST00000380333 | carbonic anhydrase VB pseudogene 1 | 1.16E-02 | 1.530 |
| MTF1 | BC014454 | metal-regulatory transcription factor 1 | 1.70E-03 | 1.526 |
| LRP1 | ENST00000243077 | low density lipoprotein receptor-related protein 1 | 1.89E-03 | 1.525 |
| LSP1 | AK290279 | lymphocyte-specific protein 1 | 2.51E-03 | 1.524 |
| NCSTN | AF240468 | nicastrin | 5.27E-04 | 1.524 |
| ABCA1 | AF285167 | ATP-binding cassette, sub-family A (ABC1), member 1 | 2.80E-02 | 1.523 |
| BEST1 | AF057170 | bestrophin 1 | 2.53E-03 | 1.520 |
| FZD5 | ENST00000295417 | frizzled family receptor 5 | 3.01E-03 | 1.520 |
| G6PC3 | BC021574 | glucose 6 phosphatase, catalytic, 3 | 3.37E-03 | 1.519 |
| SIRPD | AK093083 | signal-regulatory protein delta | 3.25E-03 | 1.517 |
| CSF2RA | BC071835 | colony stimulating factor 2 receptor, alpha, low-affinity (granulocyte-macrophage) | 1.60E-03 | 1.517 |
| SDC3 | AB007937 | syndecan 3 | 2.20E-04 | 1.515 |
| CD93 | U94333 | CD93 molecule | 3.19E-03 | 1.515 |
| NPL | AY336748 | N-acetylneuraminate pyruvate lyase (dihydrodipicolinate synthase) | 1.80E-03 | 1.514 |
| MLF2 | AF070539 | myeloid leukemia factor 2 | 7.08E-04 | 1.513 |
| SEMA4A | AY358531 | sema domain, immunoglobulin domain (Ig), transmembrane domain (TM) and short cytoplasmic domain, (semaphorin) 4A | 3.74E-03 | 1.511 |
| CCR2 | U03882 | chemokine (C-C motif) receptor 2 | 2.29E-02 | 1.511 |
| ARHGEF11 | BC057394 | Rho guanine nucleotide exchange factor (GEF) 11 | 1.56E-03 | 1.509 |
| NFIA | AK299289 | nuclear factor I | 4.03E-03 | 1.508 |
| LINC00537 | BC110369 | long intergenic non-protein coding RNA 537 | 8.22E-03 | 1.507 |
| P2RY2 | BC012104 | purinergic receptor P2Y, G-protein coupled, 2 | 1.54E-02 | 1.504 |
| EREG | D30783 | epiregulin | 3.45E-02 | 1.504 |
| NLRC4 | AF376061 | NLR family, CARD domain containing 4 | 7.58E-03 | 1.502 |
| MLLT3 | ENST00000380338 | myeloid | 2.21E-02 | -1.502 |
| CD28 | J02988 | CD28 molecule | 1.69E-02 | -1.504 |
| STAT4 | AK299976 | signal transducer and activator of transcription 4 | 1.49E-02 | -1.504 |
| RN5S196 | ENST00000516984 | RNA, 5S ribosomal 196 | 4.76E-02 | -1.505 |
| FLJ38379 | AK095698 | uncharacterized FLJ38379 | 2.84E-03 | -1.506 |
| SNORD94 | NR_004378 | small nucleolar RNA, C | 5.70E-03 | -1.508 |
| JAKMIP2 | AB011127 | janus kinase and microtubule interacting protein 2 | 4.28E-02 | -1.510 |
| TSPYL1 | NM_003309 | TSPY-like 1 | 4.03E-03 | -1.512 |
| RHOH | BC014261 | ras homolog family member H | 1.21E-02 | -1.514 |
| ETS1 | BX640634 | v-ets erythroblastosis virus E26 oncogene homolog 1 (avian) | 3.97E-03 | -1.515 |
| RNU5A-1 | NR_002756 | RNA, U5A small nuclear 1 | 3.02E-02 | -1.522 |
| SOD1 | EF151142 | superoxide dismutase 1, soluble | 1.18E-03 | -1.524 |
| PTGES3P1 | ENST00000439531 | prostaglandin E synthase 3 (cytosolic) pseudogene 1 | 1.50E-02 | -1.526 |
| FAM46C | BC131726 | family with sequence similarity 46, member C | 3.42E-02 | -1.529 |
| STAT1 | M97935 | signal transducer and activator of transcription 1, 91kDa | 9.22E-03 | -1.532 |
| SLFN13 | BC136622 | schlafen family member 13 | 1.73E-03 | -1.532 |
| CPA3 | BC012613 | carboxypeptidase A3 (mast cell) | 2.37E-02 | -1.536 |
| TGFBR3 | AK291345 | transforming growth factor, beta receptor III | 2.85E-02 | -1.537 |
| FAM169A | AK290004 | family with sequence similarity 169, member A | 1.93E-02 | -1.537 |
| SNORD4B | NR_000009 | small nucleolar RNA, C | 1.05E-03 | -1.538 |
| INPP4B | BC110918 | inositol polyphosphate-4-phosphatase, type II, 105kDa | 1.08E-02 | -1.538 |
| MCOLN2 | BC104891 | mucolipin 2 | 8.75E-03 | -1.540 |
| MYO6 | ENST00000369977 | myosin VI | 3.21E-02 | -1.541 |
| EPB41L4A | AB030240 | erythrocyte membrane protein band 4.1 like 4A | 4.95E-02 | -1.541 |
| ODF2L | AB033055 | outer dense fiber of sperm tails 2-like | 1.98E-02 | -1.546 |
| SNORD38B | ENST00000484599 | small nucleolar RNA, C | 2.06E-03 | -1.547 |
| IL18RAP | AF077346 | interleukin 18 receptor accessory protein | 3.52E-02 | -1.547 |
| THEMIS | AK128377 | thymocyte selection associated | 3.61E-02 | -1.550 |
| ABCA5 | AY028897 | ATP-binding cassette, sub-family A (ABC1), member 5 | 2.01E-03 | -1.551 |
| EIF4A2 | AK296279 | eukaryotic translation initiation factor 4A2 | 4.40E-03 | -1.552 |
| CCR4 | BC071751 | chemokine (C-C motif) receptor 4 | 2.21E-02 | -1.552 |
| TRNAU2 | ENST00000466665 | transfer RNA selenocysteine 2 (anticodon UCA) | 4.69E-02 | -1.555 |
| KIF21A | AY368076 | kinesin family member 21A | 7.75E-03 | -1.556 |
| CATSPERB | AK126034 | catsper channel auxiliary subunit beta | 8.18E-03 | -1.556 |
| SNORD64 | NR_001294 | small nucleolar RNA, C | 1.27E-02 | -1.566 |
| SNORA22 | NR_002961 | small nucleolar RNA, H | 1.89E-02 | -1.568 |
| CLEC2D | BC019883 | C-type lectin domain family 2, member D | 1.16E-02 | -1.569 |
| SNORD42A | NR_000014 | small nucleolar RNA, C | 1.14E-02 | -1.571 |
| XCL1 | D43768 | chemokine (C motif) ligand 1 | 2.96E-02 | -1.572 |
| MGC2752 | BC065480 | CENPB DNA-binding domains containing 1 pseudogene | 5.63E-03 | -1.574 |
| TTC39B | AK093459 | tetratricopeptide repeat domain 39B | 8.68E-03 | -1.576 |
| SNORA24 | NR_002963 | small nucleolar RNA, H | 4.13E-02 | -1.576 |
| SCARNA5 | NR_003008 | small Cajal body-specific RNA 5 | 4.31E-03 | -1.578 |
| NPCDR1 | AF156973 | nasopharyngeal carcinoma, down-regulated 1 | 2.50E-02 | -1.581 |
| KLF12 | AF330041 | Kruppel-like factor 12 | 1.13E-03 | -1.583 |
| NCALD | AF251061 | neurocalcin delta | 1.11E-02 | -1.587 |
| SNHG3 | AK092726 | small nucleolar RNA host gene 3 (non-protein coding) | 4.66E-02 | -1.593 |
| MAPKAPK5-AS1 | AK096935 | MAPKAPK5 antisense RNA 1 (non-protein coding) | 3.41E-03 | -1.596 |
| SCARNA10 | NR_004387 | small Cajal body-specific RNA 10 | 7.39E-03 | -1.597 |
| TMEM14A | BC015097 | transmembrane protein 14A | 3.55E-03 | -1.597 |
| SNORD58A | NR_002571 | small nucleolar RNA, C | 1.74E-02 | -1.604 |
| TSPAN2 | BC021675 | tetraspanin 2 | 1.93E-03 | -1.610 |
| PRSS23 | BC001278 | protease, serine, 23 | 1.15E-02 | -1.611 |
| GAS5 | BC038733 | growth arrest-specific 5 (non-protein coding) | 2.76E-02 | -1.611 |
| SNORD14D | ENST00000527983 | small nucleolar RNA, C | 2.21E-02 | -1.611 |
| SNORD1A | NR_004395 | small nucleolar RNA, C | 1.51E-03 | -1.612 |
| NELL2 | AK295125 | NEL-like 2 (chicken) | 1.89E-02 | -1.613 |
| KPNA5 | BC047409 | karyopherin alpha 5 (importin alpha 6) | 9.12E-04 | -1.618 |
| KLRB1 | AK292022 | killer cell lectin-like receptor subfamily B, member 1 | 2.83E-02 | -1.628 |
| CXorf65 | ENST00000374251 | chromosome X open reading frame 65 | 2.61E-03 | -1.632 |
| SNHG12 | AK092096 | small nucleolar RNA host gene 12 (non-protein coding) | 4.59E-03 | -1.642 |
| GBP4 | AF288814 | guanylate binding protein 4 | 3.22E-03 | -1.642 |
| SNHG1 | AK095849 | small nucleolar RNA host gene 1 (non-protein coding) | 2.27E-02 | -1.645 |
| GCNT4 | AF132035 | glucosaminyl (N-acetyl) transferase 4, core 2 | 2.39E-02 | -1.645 |
| FGFBP2 | BC025720 | fibroblast growth factor binding protein 2 | 4.85E-02 | -1.646 |
| SNORD30 | NR_002561 | small nucleolar RNA, C | 1.45E-02 | -1.647 |
| KLRF1 | AF175206 | killer cell lectin-like receptor subfamily F, member 1 | 3.41E-02 | -1.651 |
| SNORA3 | ENST00000530585 | small nucleolar RNA, H | 1.48E-02 | -1.652 |
| DOCK9 | AB028981 | dedicator of cytokinesis 9 | 2.72E-03 | -1.656 |
| GZMA | BC015739 | granzyme A (granzyme 1, cytotoxic T-lymphocyte-associated serine esterase 3) | 2.98E-02 | -1.660 |
| SNORD81 | ENST00000363840 | small nucleolar RNA, C | 8.22E-03 | -1.668 |
| SYNE2 | AF435011 | spectrin repeat containing, nuclear envelope 2 | 3.27E-03 | -1.672 |
| SNORA68 | NR_000012 | small nucleolar RNA, H | 1.73E-02 | -1.673 |
| GBP5 | AF430642 | guanylate binding protein 5 | 5.01E-03 | -1.687 |
| SAMD3 | AK302228 | sterile alpha motif domain containing 3 | 1.38E-02 | -1.714 |
| CEP78 | BC128058 | centrosomal protein 78kDa | 4.02E-03 | -1.747 |
| ABCD2 | BC104901 | ATP-binding cassette, sub-family D (ALD), member 2 | 7.35E-03 | -1.762 |
| GPR174 | BC104922 | G protein-coupled receptor 174 | 5.36E-03 | -1.789 |
| SNORD14E | NR_003125 | small nucleolar RNA, C | 1.72E-03 | -1.812 |
| TIGIT | BC101288 | T cell immunoreceptor with Ig and ITIM domains | 2.74E-03 | -1.817 |
| SNORD28 | NR_002562 | small nucleolar RNA, C | 6.55E-03 | -1.822 |
| IKZF2 | AF130863 | IKAROS family zinc finger 2 (Helios) | 1.02E-02 | -1.824 |
| TAF1D | AK128061 | TATA box binding protein (TBP)-associated factor, RNA polymerase I | 1.36E-03 | -1.841 |
| DTHD1 | AK304357 | death domain containing 1 | 7.60E-03 | -1.843 |
| SNHG16 | BC042949 | small nucleolar RNA host gene 16 | 4.67E-03 | -1.856 |
| SNORD116-24 | NR_003338 | small nucleolar RNA, C | 1.67E-02 | -1.859 |
| SNORD54 | X96653 | small nucleolar RNA, C | 5.57E-03 | -1.868 |
| SCARNA6 | NR_003006 | small Cajal body-specific RNA 6 | 4.61E-03 | -1.905 |
| HRH4 | AF312230 | histamine receptor H4 | 7.81E-03 | -1.932 |
| RPL13A | AK056837 | ribosomal protein L13a | 6.57E-03 | -1.947 |
| C12orf75 | BC013920 | chromosome 12 open reading frame 75 | 4.89E-03 | -1.949 |
| KLRC3 | AF078550 | killer cell lectin-like receptor subfamily C, member 3 | 4.12E-02 | -1.950 |
| RBMX | ENST00000565907 | RNA binding motif protein, X-linked | 2.81E-03 | -2.031 |
| MS4A3 | L35848 | membrane-spanning 4-domains, subfamily A, member 3 (hematopoietic cell-specific) | 6.61E-03 | -2.056 |
| KLRC4 | AF350018 | killer cell lectin-like receptor subfamily C, member 4 | 2.94E-02 | -2.144 |
| KLRC4-KLRK1 | AF461811 | KLRC4-KLRK1 readthrough | 2.30E-02 | -2.232 |
| SNORA20 | NR_002960 | small nucleolar RNA, H | 7.17E-03 | -2.272 |
| SNORD45C | ENST00000496055 | small nucleolar RNA, C | 6.01E-03 | -2.281 |
| GZMK | BC035802 | granzyme K (granzyme 3; tryptase II) | 1.97E-02 | -2.408 |
| UTS2 | BC126443 | urotensin 2 | 2.19E-02 | -2.484 |
| CLC | L01664 | Charcot-Leyden crystal protein | 1.22E-02 | -3.162 |
