# Supplementary material for: Gene expression profiling reveals potential prognostic biomarkers associated with the progression of heart failure
Source: Genome Med. 2015 Mar 14;7(1):26. doi: 10.1186/s13073-015-0149-z (PMC4432772; doi:10.1186/s13073-015-0149-z)
Supplement: Additional file 10: — Differentially expressed genes in non-HF patients versus control patients. [file 13073_2015_149_MOESM10_ESM.doc]

**Additional file** **10.** Differentially expressed genes in non-HF patients versus control patients

| **Gene Symbol** | **RefSeq** | **Gene assignment** | ***p*-value** | **Fold change** |
| --- | --- | --- | --- | --- |
| GPR15 | BC101779 | G protein-coupled receptor 15 | 2.34E-02 | 2.818 |
| GSTM1 | J03817 | glutathione S-transferase mu 1 | 1.60E-02 | 2.703 |
| HP | L29394 | haptoglobin | 6.75E-03 | 2.609 |
| LRRN3 | BC035133 | leucine rich repeat neuronal 3 | 1.04E-02 | 2.529 |
| ECRP | NR_033909 | ribonuclease, RNase A family, 2 (liver, eosinophil-derived neurotoxin) pseudogene | 7.98E-03 | 2.181 |
| SASH1 | ENST00000367467 | SAM and SH3 domain containing 1 | 1.80E-03 | 1.910 |
| RNASE2 | M28129 | ribonuclease, RNase A family, 2 (liver, eosinophil-derived neurotoxin) | 2.56E-02 | 1.891 |
| PPARG | ENST00000397010 | peroxisome proliferator-activated receptor gamma | 1.59E-02 | 1.780 |
| AQP9 | AF016495 | aquaporin 9 | 1.04E-02 | 1.779 |
| CD24 | NM_013230 | CD24 molecule | 1.47E-02 | 1.766 |
| DSC2 | BC063291 | desmocollin 2 | 1.28E-02 | 1.723 |
| ASGR2 | AF529374 | asialoglycoprotein receptor 2 | 3.82E-03 | 1.685 |
| STEAP4 | AF423422 | STEAP family member 4 | 1.67E-02 | 1.674 |
| SERPINB10 | BC096217 | serpin peptidase inhibitor, clade B (ovalbumin), member 10 | 3.54E-02 | 1.663 |
| CD180 | D83597 | CD180 molecule | 1.23E-03 | 1.658 |
| FCGR1A | AK291502 | Fc fragment of IgG, high affinity Ia, receptor (CD64) | 1.79E-02 | 1.644 |
| CD22 | AK301177 | CD22 molecule | 9.06E-03 | 1.642 |
| FAM198B | BC043193 | family with sequence similarity 198, member B | 4.45E-03 | 1.605 |
| CR1 | ENST00000367049 | complement component (3b/4b) receptor 1 (Knops blood group) | 2.23E-02 | 1.604 |
| STAB1 | AB052956 | stabilin 1 | 9.94E-03 | 1.602 |
| CD79A | AK223371 | CD79a molecule, immunoglobulin-associated alpha | 3.64E-03 | 1.562 |
| MERTK | BC114918 | c-mer proto-oncogene tyrosine kinase | 4.85E-02 | 1.559 |
| DYSF | ENST00000409366 | dysferlin | 2.72E-02 | 1.542 |
| CR1L | BC109190 | complement component (3b/4b) receptor 1-like | 1.38E-02 | 1.534 |
| LGALS9 | AK126017 | lectin, galactoside-binding, soluble, 9 | 2.25E-02 | 1.520 |
| KIAA0226L | AK093073 | KIAA0226-like | 1.52E-03 | 1.517 |
| FCRL5 | AF343664 | Fc receptor-like 5 | 2.15E-02 | 1.501 |
| STON2 | AF449430 | stonin 2 | 1.16E-02 | -1.504 |
| CTTN | AK291097 | cortactin | 4.36E-02 | -1.511 |
| PDGFD | BC030645 | platelet derived growth factor D | 3.39E-02 | -1.523 |
| CEP78 | BC128058 | centrosomal protein 78kDa | 1.97E-02 | -1.529 |
| PDZK1IP1 | BC012303 | PDZK1 interacting protein 1 | 4.69E-02 | -1.548 |
| SNORD1A | NR_004395 | small nucleolar RNA, C | 2.95E-03 | -1.549 |
| STOM | AK304449 | stomatin | 1.45E-03 | -1.554 |
| LIPH | AY093498 | lipase, member H | 3.06E-02 | -1.555 |
| C12orf75 | BC013920 | chromosome 12 open reading frame 75 | 4.09E-02 | -1.557 |
| ENDOD1 | ENST00000278505 | endonuclease domain containing 1 | 2.18E-02 | -1.563 |
| CYP4F22 | BC069351 | cytochrome P450, family 4, subfamily F, polypeptide 22 | 3.07E-03 | -1.587 |
| GGTA1P | AF305838 | glycoprotein, alpha-galactosyltransferase 1 pseudogene | 3.67E-02 | -1.587 |
| VTRNA1-1 | NR_026703 | vault RNA 1-1 | 3.56E-02 | -1.597 |
| PLOD2 | AK295084 | procollagen-lysine, 2-oxoglutarate 5-dioxygenase 2 | 4.32E-02 | -1.597 |
| NCKAP1 | AB011159 | NCK-associated protein 1 | 2.01E-02 | -1.631 |
| EGF | AK299306 | epidermal growth factor | 3.89E-02 | -1.650 |
| PKHD1L1 | AY219181 | polycystic kidney and hepatic disease 1 (autosomal recessive)-like 1 | 3.63E-02 | -1.651 |
| G0S2 | BC009694 | G0/G1 switch 2 | 1.26E-02 | -1.651 |
| EGR1 | M62829 | early growth response 1 | 3.19E-02 | -1.702 |
| HEMGN | AY244805 | hemogen | 2.20E-02 | -1.709 |
| DENND2C | BX649075 | DENN | 1.99E-02 | -1.712 |
| FSTL1 | BC000055 | follistatin-like 1 | 1.24E-02 | -1.789 |
| PTGS2 | AY151286 | prostaglandin-endoperoxide synthase 2 (prostaglandin G/H synthase) | 4.75E-02 | -1.803 |
| TRGV9 | ENST00000444775 | T cell receptor gamma variable 9 | 1.72E-02 | -1.810 |
| PROS1 | M15036 | protein S (alpha) | 2.83E-02 | -1.853 |
| PF4 | M25897 | platelet factor 4 | 4.60E-02 | -1.906 |
| BEND2 | BC037301 | BEN domain containing 2 | 4.66E-02 | -1.916 |
| MOP-1 | AB014771 | MOP-1 | 4.47E-02 | -1.980 |
| IL8 | M17017 | interleukin 8 | 3.53E-02 | -2.026 |
| FOSB | BC036724 | FBJ murine osteosarcoma viral oncogene homolog B | 4.46E-02 | -2.071 |
| NR4A2 | AK291456 | nuclear receptor subfamily 4, group A, member 2 | 1.67E-02 | -2.280 |
