# Supplementary figures and images for: Gene expression profiling reveals potential prognostic biomarkers associated with the progression of heart failure
Source: Genome Med. 2015 Mar 14;7(1):26. doi: 10.1186/s13073-015-0149-z (PMC4432772; doi:10.1186/s13073-015-0149-z)

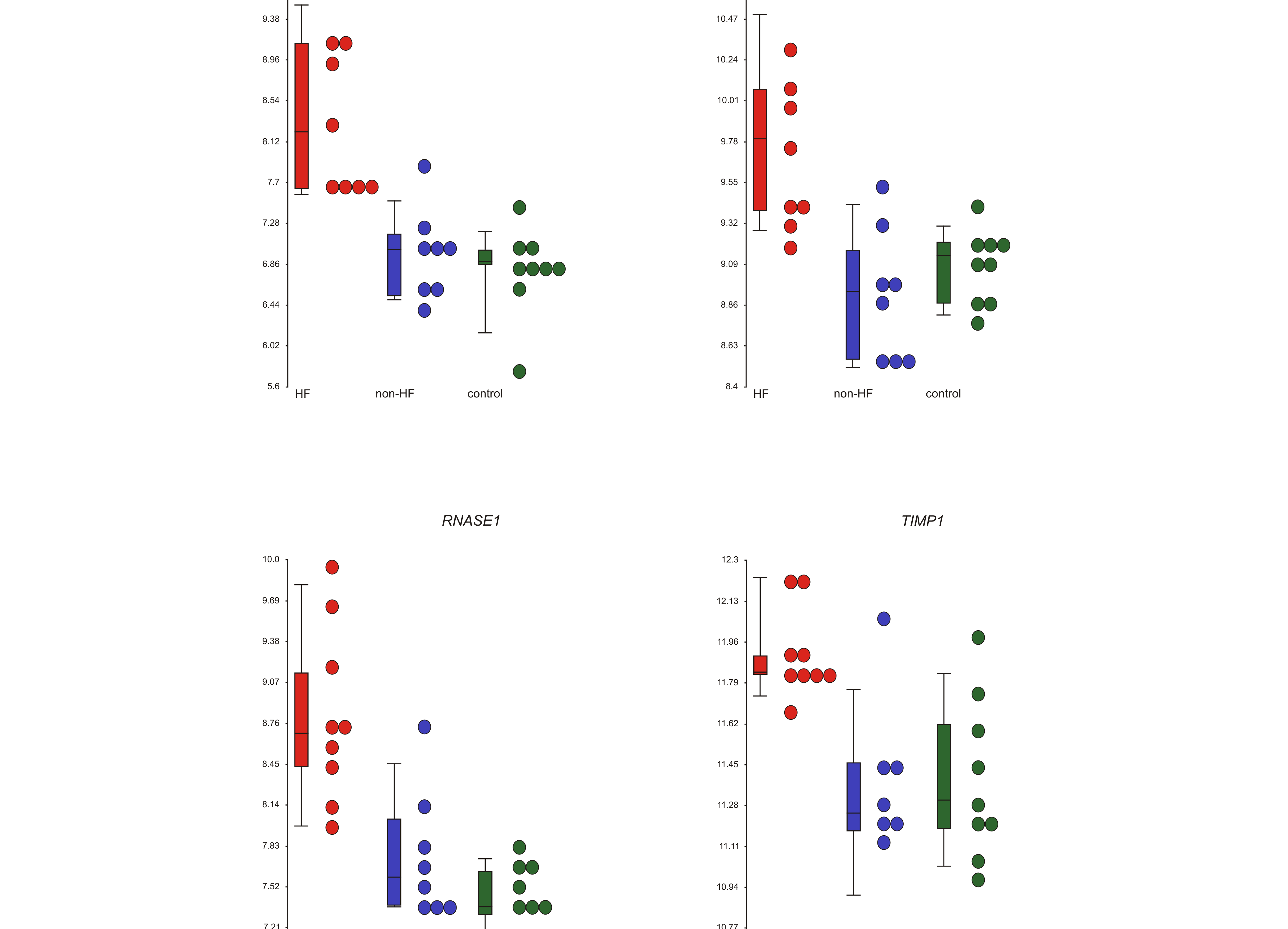

Supplement: Additional file 11: — Expression data from microarray experiments for FMN1 , JDP2 , RNASE1 , and TIMP1 at admission. The y-axis represents log2 normalized intensity of gene expression and the x-axis represents analyzed groups. The line inside the box represents the median of the samples in a group. Dots represent relative expression levels in individual HF (red), non-HF (blue), and control group patients (green). [file 13073_2015_149_MOESM11_ESM.tiff]
